# Mode-locked optomechanical frequency combs in a graphene-silica microresonator

**DOI:** 10.1126/sciadv.ady1279

**Published:** 2025-10-22

**Authors:** Hao Zhang, Yu-Pei Liang, Teng Tan, Shi-Da Wen, Yan Yu, Ning An, Yan-Wu Liu, Yan-Hong Guo, Qi-Huang Gong, Yong-Jun Huang, Yun-Jiang Rao, Yun-Feng Xiao, Qi-Fan Yang, Bai-Cheng Yao

**Affiliations:** ^1^ Key Laboratory of Optical Fibre Sensing and Communications (Education Ministry of China), University of Electronic Science and Technology of China, Chengdu, China.; ^2^ State Key Laboratory for Mesoscopic Physics and Frontiers Science Center for Nano-optoelectronics, School of Physics, Peking University, Beijing, China.; ^3^ T. J. Watson Laboratory of Applied Physics, California Institute of Technology, Pasadena, CA, USA.; ^4^ Engineering Center of Integrated Optoelectronic & Radio Meta-chips, University of Electronic Science and Technology of China, Chengdu, China.

## Abstract

Mode locking is an essential process through which resonant modes achieve stable synchronization via nonlinear interactions. This self-organization allows photonic and electronic sources to produce pulsed waveforms and is vital for applications in ultrafast and high-field optics as well as frequency comb generation. Here, we report a mechanism to include photon-electron-phonon interactions, demonstrating the excitation of mode-locked optomechanical microcombs in a graphene-deposited silica microresonator, determined by the synergy of optomechanical back action and graphene saturable absorption. The circulating optical field induces mechanical oscillations that modulate the light wave, while Pauli blocking in graphene locks a single optomechanical mode, forming a localized coherent optical wave packet within a single microcavity. In addition, using frequency division techniques, the mode-locked optomechanical microcomb achieves repetition stability with phase noise reduced to −110.5 decibels relative to the carrier per hertz at a 1-hertz offset and an Allan deviation as low as 3 × 10^−12^ @ 20 seconds, comparable to a standard rubidium clock.

## Teaser

Graphene-assisted optomechanical mode locking yields ultrastable frequency combs with atomic clock–level precision.

## INTRODUCTION

Cavity optomechanics (*1*, *2*), emerging from the interaction between electromagnetic radiation and micro/nanomechanical motion, have attracted extensive attention in science and technologies ranging from quantum technologies (*3*, *4*), communications (*5*, *6*), high-precision sensing (*7*–*9*), gyroscopy, and metrology (*10*, *11*). In the blue-detuned region of a cavity resonance, photons can emit phonons into mechanical modes, offering the mechanical gain. This creates versatile phenomena such as laser excitation (*12*, *13*), chaos generation (*14*–*16*), and acoustic comb formation (*17*–*20*). When realizing a delicate balance between mechanical dispersion and nonlinearity and between phonon gains and losses, a dissipative acoustic soliton can imprint its profile on the optical field (*21*), suggesting a possibility of optomechanical mode locking. However, similar to the formation of dissipative Kerr solitons in an optical microcavity (*22*), the dispersion-nonlinearity balance is necessary; thus, the existence of mechanical dispersion is imperative in the formation of mechanical solitons (*23*), proposing an extra requirement on the cavity structure. Meanwhile, delicate frequency detuning and precise thermal control are required, which limits the success rate of the locking formation and the quality of the pulse dynamics (*24*–*26*).

To facilitate reliable mode locking, a promising way is to introduce an optoelectronic nanomaterial into the optomechanical cavity for enhancing the saturable absorption (*27*). In this regard, graphene could be a perfect candidate. Because of its unique gapless nature, graphene allows photon-electron interactions in the broadband range from ultraviolet to terahertz (*28*, *29*) and has been widely used in pulsed lasers (*30*, *31*), modulators (*32*, *33*), and nonlinear conversions (*34*–*37*). Besides, graphene also demonstrates great potential in optomechanics owing to its exceptional mechanical properties (*38*–*40*). Technically, thanks to its atomic flexibility, graphene could be naturally suitable to integrate in microresonators (*41*), achieving impressive tunability and sensitivity for light-matter interactions (*42*, *43*). Until now, the mode locking phenomenon based on graphene has typically been observed in optics, while its demonstration in microcavity optomechanics remains elusive.

Here, we investigate nonlinear optomechanics in a graphene-assisted microresonator and report the deterministic generation of mode-locked optomechanical microcombs. Periodic acoustic waves are stimulated by a 1550-nm continuous optical pumping field in whispering-gallery mode microresonators and in turn modulate the light wave. With tuning of the intracavity optical power, the intracavity field evolves from the sinusoidal state to the transition state. In this process, resonant light activates electrons in graphene and triggers interband Pauli blocking (*44*). This effect further amasses the competitive advantage accumulation of harmonic modes and lastly forms phase locking wave packets. During the evolution, we observe both a standard lotus-like shape dual-pulse spectrum and sech^2^-like shape single-pulse spectrum, with a mechanical repetition of 7.6 MHz. Such a formation of mode-locked optomechanical microcombs deterministically relies on the intracavity power, because it uniquely stems from the cross-interaction among photons, electrons, and phonons in the graphene-silica microcavity, distinct from other microcombs generated via wave mixing (*18*, *45*), Kuznetsov-Ma growth turn (*46*), Akhmediev breathers (*47*), and motion localizing (*21*). This strong nonlinear modulation synthesis enables highly coherent comb generation. After feedback stabilization, the phase noise of the first line in the mode-locked optomechanical comb approaches down to −110.5 dBc/Hz @ 1 Hz, and the uncertainty of its frequency reaches 3 × 10^−12^ at 20 s.

## RESULTS

**Fig. 1.**
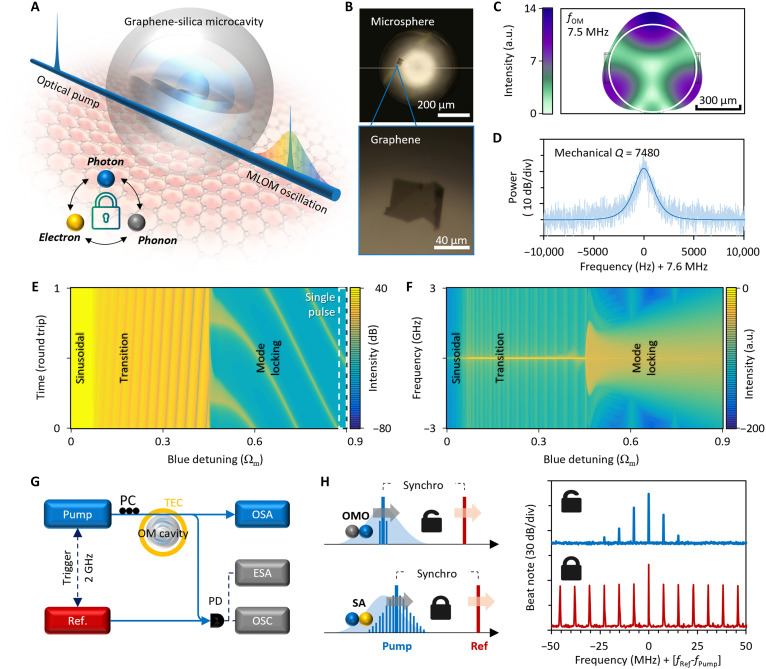
Conceptual design and mechanism of the optomechanical mode in a graphene-assisted silica microresonator. (**A**) Schematic diagram. By detuning a pump laser into an optical mode, the increasing intracavity optical power excites optomechanical oscillations via “photon-phonon” interactions, and then the optical field is modulated by mechanical oscillation and is locked by the graphene because of the blocking of “photon-electron” interaction. MLOM, mode-locked optomechanical microcomb. (**B**) Device pictures. The diameter of the silica microsphere is 610 μm, and the graphene size is 60 μm by 40 μm. (**C**) Simulated oscillation of the first-order mechanical mode. a.u., arbitrary units. (**D**) Measured mechanical resonance showing a mechanical *Q* = 7480. Dark blue curve, Lorentzian fitting. (**E** and **F**) Calculated maps showing that optomechanical oscillation evolves from the sinusoidal state to the mode locking state. a.u., arbitrary units. (**G**) Experimental setup for generating and measuring the optomechanical combs. PC, polarization controller; PD, photodetector; TEC, thermo-electric cooler; OSA, optical spectrum analyzer; ESA, electrical spectrum analyzer; OSC, oscilloscope. (**H**) Detuning scheme and measured optomechanical oscillation before and after mode locking. OMO, optomechanical oscillation; SA, saturable absorption.

Figure 1A shows the conceptual design of our graphene-assisted silica microresonator for generating mode-locked optomechanical microcombs. A silica microsphere with a diameter of ≈690 μm [free spectral range (FSR) ≈ 95.2 GHz] supports both high–quality (*Q*) factor optical and mechanical modes. Subsequently, we deposit a mechanically exfoliated monolayer graphene flake by using the dry-transfer technique (*48*). The location of the graphene nanosheet is on 10° above the equatorial plane for ensuring effective light-material interaction while avoiding heating damage. When tuning a single-frequency pump laser into a resonance of the microcavity (from the blue-detuned side) via a tapered fiber, we can obtain optomechanical oscillations as optical energy is fed into the mechanical mode via the photon-phonon transition. When the intracavity power meets the saturable absorption condition of graphene, modulation between the light wave and the mechanical wave will trigger the electron-photon interaction on the basis of Pauli blocking, leading to mode locking output. We show the detailed theoretical analysis in note S1.

Figure 1B illustrates the optical microscope images, in which a monolayer graphene with a size of 60 μm by 40 μm is located close to the equator of the microcavity. Determined by the silica microsphere material and geometry, the fundamentally oscillating frequency of the mechanical breathing mode is 7.6 MHz. The intrinsic mechanical oscillation field is simulated in Fig. 1C. Here, the white circle marks the contour of our microsphere cavity. Such a mechanical oscillation can be directly stimulated by radiation pressure force exerted by the intracavity field. When the intracavity optical power is about to reach the optomechanical oscillation threshold, the mechanical mode could be observed in radio frequency (RF), as plotted in Fig. 1D. The linewidth of the resonant peak is 1.01 kHz after Lorentzian fitting. This suggest that the intrinsic mechanical *Q* factor of this microresonator reaches 7480. Typically, measured optical *Q* factors of this microresonator are 1.4 × 10^8^ before graphene deposition and 9 × 10^7^ after graphene deposition. A higher *Q* factor allows for the excitation of rich optomechanical harmonics using a smaller pump power, while graphene-induced absorption plays a crucial role in mode locking formation. In the meantime, we need to ensure that the graphene’s absorption is adequate for effective modulation. We provide detailed discussion, device fabrication flow, and characterizations in notes S1 and S2.

On the basis of solving the revised Heisenberg equations of motion while accounting for the saturable absorption of graphene and using the split-step method, Fig. 1 (E and F) illustrates the calculated temporal and spectral evolution of the optomechanical oscillation when the pump laser is tuned from the center of the optical resonance to the blue-detuned side. As the pumping frequency moves away from the center, the intracavity optical power gradually decreases, enhancing the saturable absorption effect in the graphene, while mechanical oscillation is amplified because of the proliferation of phonons emitted from optical mode into mechanical mode (*2*). The saturable absorption and mechanical oscillation work in synergy, modulating the intracavity field and ultimately leading to a mode locking state. In this state, the optical field in the microcavity manifests as sharp, phase-locked pulses rather than flat, incoherent waveforms. Consequently, the optical spectrum expands to several gigahertz with hundreds of coherently oscillating modes and symmetrical envelopes.

Figure 1G illustrates the experimental setup used to generate optomechanical combs and measure their time-frequency characteristics. A tunable continuous-wave laser diode serves as the pump, while a separate reference laser is used to beat with the comb. The frequency difference between the pump and reference lasers is maintained at a constant 2 GHz. This configuration enables the equivalent observation of the optical spectrum of the optomechanical oscillation after photodetection. In Fig. 1H, we demonstrate that as the pump laser is tuned, its frequency moves progressively into the deeply blue-detuned region of an optical resonance. This results in a growth of the optomechanical oscillation amplitude despite a decrease in the intracavity optical power, forming the locking state. This figure also shows the measured optomechanical oscillation spectra before and after mode locking. A substantial spectral broadening is clearly observed after the onset of mode locking.

**Fig. 2.**
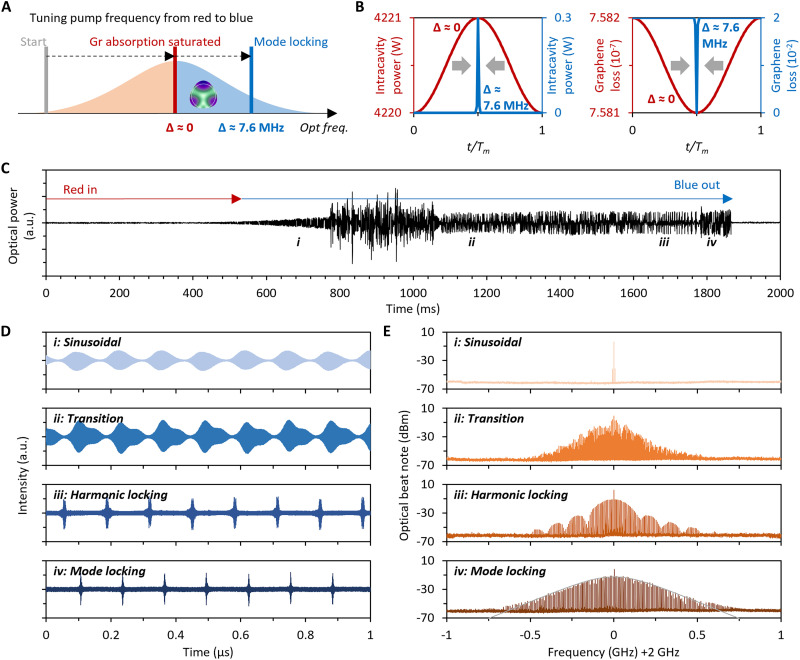
Optomechanical evolution and time-frequency measurements. (**A**) Generation scheme of our optomechanical mode locking in a microcavity resonance. (**B**) Calculated intracavity optical power and graphene-induced loss when Δ values are 0 and 7.6 MHz (blue detuned). (**C**) Evolution of the optomechanical oscillation in a resonance located around 1550.321 nm. The optomechanical wave evolves from the periodic state and transition state to dual-pulse and single-pulse mode locking states (i to iv). (**D** and **E**) Temporal and spectral measurements in state i to state iv. When mode locking, the resonant mechanical waves present comb-like spectra corresponding to stable pulses with a repetition of 7.6 MHz and a pulse period of 131.58 ns. a.u., arbitrary units.

Figure 2A illustrates our method for initiating mode locking in the microcavity schematically. We tune the pump laser from red to blue, stopping it in a blue-detuned region away from the center when reaching the mode locking state. This operation enables stable pulse generation through periodic intensity modulation, relying on optomechanical coupling and the saturable absorption properties of graphene. During this process, we first ensure sufficiently high intracavity power (red line). In this state, intracavity power is much higher than the saturation power of graphene; thus, the graphene’s absorption is well saturated and cannot modulate the optical wave through Pauli blocking. We then gradually adjust the pump frequency toward the blue-detuned region. During this adjustment, the detuning increases and the intracavity optical power decreases; both the mechanical oscillation and saturable absorption become more pronounced, thereby enhancing the modulation depth of the intracavity field. Once the intensity modulation is sufficiently effective, mode-locked pulses are generated (blue line). Given that optomechanical oscillations occur in the blue-detuned region, establishing and maintaining the optomechanical mode locking state would not be affected by the thermal instability of this microresonator associated with the red-detuned region. In Fig. 2B, we present the calculated intracavity power and graphene-induced loss with varying detuning. As the detuning changes from 0 to 7.6 MHz, the maximum graphene-induced loss increases from 7.852 × 10^−7^ to 2 × 10^−2^, establishing the modulation. Consequently, the minimum intracavity power decreases from 4220 W to less than 1 μW, significantly suppressing the dc component. More measured results are shown in note S3.

Figure 2C demonstrates the measured evolution trace of the microcavity’s output. We adjust the pump from the red side into resonance and then excite optomechanical oscillations in the blue-detuned region. During blue detuning, state transitions of the intracavity field can be well observed through the transmission. We start scanning the pumping frequency at a wavelength of 1550.321 nm from red to blue. The scanning speed of the pump frequency is 0.25 GHz/s, and the scanning period is 2 s. This scanning range is adequate to cover the effective detuning change in a range of 0 to 7.6 MHz in the presence of thermal shift. During pump scanning, the optomechanical oscillation traverse four states in sequence, from sinusoidal, transition, and harmonic locking to mode locking (i to iv). Specifically, when the pump is close to the resonance center, a sinusoidal waveform is excited owing to optomechanical coupling (state i). Then, a further increment in detuning induces cascaded mechanical sidebands around the optical signal in the frequency domain because of enhanced optomechanical oscillation amplification and stronger optical modulation from graphene (state ii) (*21*, *49*). When the intracavity modulation depth is high enough (e.g., 2%), temporal peaks with a very weak dc component are formed, the system enters a regime in which only coherent pulses remain (states iii and iv), and typically, single-pulse mode locking requires a lower intracavity power than harmonic locking (*50*). Last, once the pump frequency enters the very far blue-detuned region of the resonance, optomechanical oscillation will quickly annihilate because the intracavity power becomes below the optomechanical oscillation threshold.

Through holographic data collection, we demonstrate the temporal traces and spectra of these four states in Fig. 2 (D and E). The measured traces not only contain the slow mechanical oscillation envelopes but also keep the frequency difference between the pump and a reference laser (2 GHz). We show the experimental setup in note S3. Specifically, in the dual-pulse state (iii), we see a lotus-like spectrum, which exhibits typical characteristics of two-pulse interference. Meanwhile, in the single-pulse state (iv), the device outputs a quasi-sech^2^ shape coherent comb spectrum in the frequency domain, with a stable 7.6-MHz interval. The 3-dB bandwidth of the mode-locked optomechanical spectrum reaches ≈0.18 GHz. Such a phenomenon is notably similar to an optical frequency comb based on mode locking (*24*), which verifies that the pulse shape is constrained by the gain-loss balance. According to the spectrum of state iv (mode locking state) in Fig. 2E, the total power of non–pump frequency components is 1.63 mW, while the total power of the optomechanical comb (including the pump) is 2.24 mW, of which 72.67% is contributed by new frequency components, hinting at relatively high conversion efficiency. Typically, the efficiency of the optomechanical comb formation is determined by the optomechanical coupling rate, which can be increased by reducing the cavity size (*1*). We note that in the same silica microcavity without graphene coverage, one cannot see the mode locking operation, because the “photon-phonon-electron”–based pairwise interactions are indispensable. We show the measured results in note S3 and display the related simulations in note S1. In addition, our measurements indicate that during mode-locked optomechanical comb formation, other optical nonlinear effects such as four-wave mixing or Raman scattering have a minimal impact. Given that optomechanics can be considered a form of cubic nonlinearity (*20*), it has the potential to interact and compete with other nonlinear processes. More analyses are shown in note S1.

**Fig. 3.**
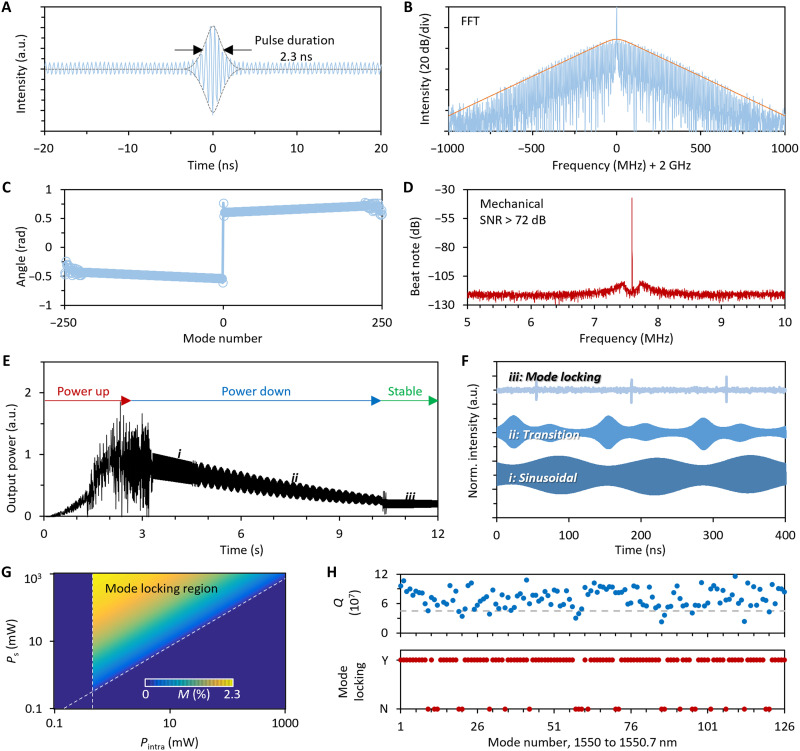
Characterization of the optomechanical mode locking and its deterministic generation. (**A**) The measured single pulse of the mode locking state contains the pump-reference beating oscillation inside. Pulse duration, 2.3 ns. (**B**) Spectrum retrieved from the measured pulse train based on the fast Fourier transform (FFT). Orange curve, envelope fitting. (**C**) Phase spectrum. It indicates that there exists chirping in the pulse train. (**D**) A measured window zooms the self-beating signal (@7.6 MHz) in. The signal-to-noise ratio (SNR) of this signal is >72 dB. Bandwidth, 1 kHz. (**E**) Formation of the optomechanical mode locking state by fixing the pump frequency while increasing the pump power. The mode locking state (iii) appears after sinusoidal (i) and transition (ii) states subsequently. (**F**) Temporal traces of the five states. The optomechanical frequency remains at 7.6 MHz. (**G**) Calculated modulation depth based on saturable absorption for mode locking formation. (**H**) Optomechanical mode locking possibility for all the 126 modes in one single microresonator in the optical range of 1550 to 1550.7 nm. Blue dots, *Q* factors of different resonances; red dots, yes (Y)/no (N) of optomechanical locking. a.u., arbitrary units.

In Fig. 3, we characterize the mode locking performance and show the deterministic generation of optomechanical mode locking. In Fig. 3A, we zoom in on a single pulse of state iv, as shown in Fig. 2C. This intuitively shows the pulse duration and internal structure of the measured pulse. Here, the fast sinusoidal oscillation in pulse illustrates the frequency difference between the pump light and the reference light; it has a period of 0.5 ns. The pulse is in quasi-sech^2^ fitting (gray dashed curve) and indicates a pulse duration of 2.3 ns. This number meets the measured spectral width, satisfying the Fourier time-bandwidth product for a hyperbolic secant function. Using the Hilbert transform and Fourier transform, we can obtain the spectrum of the pulse train, as shown in Fig. 3B. The retrieved spectrum also shows a 3-dB bandwidth of ≈0.18 GHz and a repetition rate of 7.6 MHz, meeting the measured result well. Figure 3C displays the phase spectrum of the optomechanical comb in the mode locking state. We find that in collaborative oscillating, the phase angle of every mode number remains smooth, verifying its phase locking nature. Meanwhile, the phase spectrum is asymmetric, suggesting that there is inherent chirping inside. Such a phenomenon means that the mode locking wave is not a soliton, which relies on the nonlinear-dispersion balance (*21*). In Fig. 3D, we examine the specifics of the self-beating signal in the mode-locked state, which is directly measured using a photodetector. In experimental measurement, the first mechanical oscillation locates at ≈7.6 MHz, showing a linewidth less than 10 Hz, with a high signal-to-noise ratio (SNR) higher than 72 dB. This result suggests that the mode locking state is in high coherence.

Distinct from optomechanical soliton formation relying on double balance (*21*), in this mode locking operation, the balance of gain and loss can be achieved by matching the optical gain brought by optomechanical back action and total cavity loss. Meanwhile, there is no balance between saturable absorption–related nonlinearity and dispersion, as the span of the optomechanical comb is small. This indicates that the mode locking state can be deterministically attained by appropriately adjusting the pump power once the pump frequency is in the blue-detuned region. For further verification, we maintained a constant pump detuning (Δ ≈ 7.6 MHz) in the blue-detuned region and varied the pump power by first increasing and then decreasing it. We observed that the optomechanical oscillation can also enter the mode locking state (Fig. 3E). Similar to the detuning control method, we first ensured that the intracavity optical power was sufficiently high and then fixed the pump detuning while gradually reducing the pump power from 200 to 10 mW. During this process, the evolution of the optomechanical oscillation also evolves through states i (sinusoidal), ii (transition), and iii (mode locking). It is important to introduce a high optical power initially to induce intracavity overload, providing the initial high-intensity optomechanical oscillation necessary for subsequent mode locking. In Fig. 3F, we present the temporal traces of these three states, measured at pump powers of 173, 102, and 21 mW, with normalized intensities. The waveforms display the same characteristics as those in detuning control. This result suggests that mode-locked optomechanical oscillations achieved through both detuning adjustment and optical power manipulation are consistent, confirming that as for the formation of mode locking, the major contribution comes from the graphene-based saturable absorption.

Moreover, because of the unique pulse locking mechanism, the exciting success rate of mode locking in our graphene-assisted microcavity is independent of the optically pumping mode family. We can understand that the formation of the mode locking state for an optomechanical comb just demands the intracavity power *P*_intra_ that exceeds the optomechanical oscillation threshold, while *P*_s_ induces enough saturable absorption modulation depth. Here, *P*_s_ is the graphene’s saturation power. In Fig. 3G, we calculate the modulation depth (*M*) dependent on *P*_intra_ and *P*_s_. For a fixed pump power, *P*_intra_ scales with the optical *Q* factor, which suggests that the optomechanical mode locking state tends to appear in optical modes whose *Q* factor is sufficient. Given that our graphene-assisted microresonator supports 126 mode families, we statistically measure all their *Q* factors in the band of 1550 to 1550.7 nm, about an FSR, as shown in Fig. 3H. The average *Q* of all the resonances reaches 7.2 × 10^7^. By scanning the same pump power (200 mW) in the 126 resonances one by one, we find that the optomechanical mode locking state could be generated in 107 resonances, and the mode locking appearance ratio is 80.9%. Some of the modes cannot generate a mode-locked optomechanical comb because of their relatively low *Q* or low coupling rate. More measured data are shown in note S3. In addition, we note that the desired outcome could also be achieved by using a pump with a different wavelength. On the one hand, optomechanical mode locking relies on the manipulation of in-resonance power; on the other hand, graphene demonstrates broadband saturable absorption because of its nature as a gapless nanomaterial (*29*). In note S3, we verify that by using pumps at ~1530 and 1570 nm, the mode locking state can be effectively achieved.

**Fig. 4.**
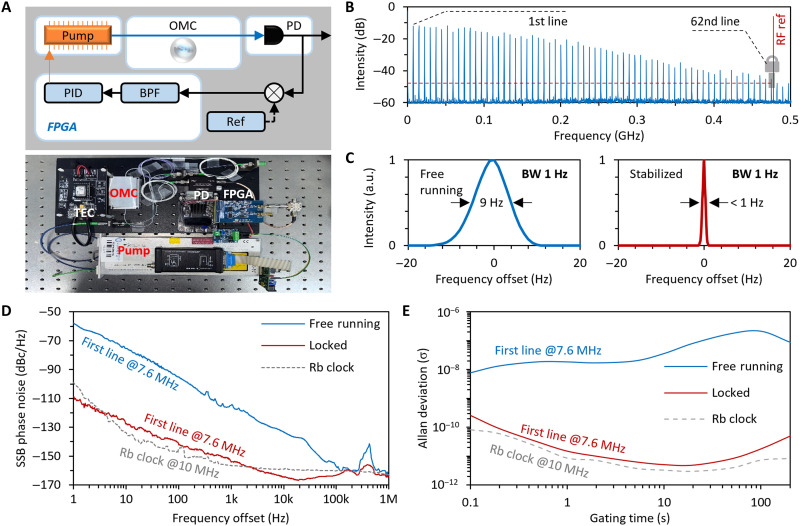
Stabilizing the mode-locked optomechanical comb. (**A**) Compact setup of the stabilized optomechanical comb. The comb lines can be locked on a radio frequency (RF) reference via an electronic feedback loop. OMC, optomechanical cavity; FPGA, field-programmable gate array; PD, photodetector; BPF, bandpass filter; PID, proportional-integral-derivative. (**B**) Scheme of the stabilization. We use a reference signal to lock the 62nd line (at 482.2 MHz) of the optomechanical comb in the spectrum. (**C**) Measured integral linewidth of the first comb line before (blue curve) and after (red curve) stabilization. BW, bandwidth. (**D** and **E**) Measured SSB-PNs and Allan deviation of the first comb line with a central frequency of 7.6 MHz. Here, blue curves show free running operation, and red curves show the case after stabilization, while gray dashed curves show the performance of a standard rubidium atomic clock.

In practice, the highly coherent mode-locked optomechanical oscillation can be used in many applications. For instance, referring the optical frequency division technique (*51*, *52*), we can achieve microwave frequency division and realize a fully stabilized optomechanical comb. This enables a way to output high-quality signals in a short-wave band, forming a compact “microwave clock.” Figure 4A demonstrates the device design. In this architecture, a pump laser, a graphene-assisted optomechanical microresonator, a temperature controller, a photodetector, and an electrical feedback loop are encapsulated in an all-in-one and plug-and-play centimeter-size module. The electronic feedback loop could be easily realized by using a field-programmable gate array (FPGA) chip, while the frequency reference could be a radio frequency generator (model: R&S SMA100B; option: SMAB-B711N). In the mode locking state, the optomechanical frequency (Ω_m_) could be adjusted through pump detuning via an optical spring effect (*2*). Figure 4B demonstrates our scheme of how to achieve an ultrastable optomechanical signal on the basis of frequency division. Data here show the measured single band spectrum of our optomechanical comb. In the mode locking state of the optomechanical comb, the frequency noise of the *N*_th_ comb line is *N*δ; here, δ is the noise of the first comb line. When we lock the *N*_th_ line on a radio frequency reference, the frequency noise of this line becomes Θ; here, Θ is the suppressed noise. Therefore, the noise of the first comb line after feedback locking becomes Θ/*N*, which could be orders lower than δ. We note that such a method of denoising low-frequency components by locking high-frequency components was mainly achieved in coherent mode-locked optomechanical combs (*53*). Here, we lock the 62nd optomechanical comb line at 471.2 MHz, with the intensity of this line measured at −45.3 dB (lockable threshold, ≈−48 dB). The frequency division ratio (*N* = 62) is primarily limited by the intensity of the comb line, as well as the trade-off between the sensitivity and the bandwidth in a photodetector. In the future, one can further enhance the frequency division ratio to reach the hundreds by using a smaller microcavity with an improved optomechanical coupling coefficient (*2*) or by leveraging thermal broadening effects (*45*). In addition, using a photodetector with both a large bandwidth and high sensitivity can also be beneficial for boosting the performance of frequency division–based stabilization.

In Fig. 4C, we show the integral linewidth at Ω_m_ before and after feedback locking. Specifically, the integral linewidth of the first comb line in the free running state is 9 Hz. However, after stabilization, the integral linewidth of the first comb line in the free running state becomes <1 Hz; this result is just limited by the resolution of our radio frequency spectrometer. For further illustrating the stability, we show the single sideband phase noise (SSB-PN) of the first comb line in Fig. 4D. In free-running operation, the SSB-PN of the mode-locked state reaches −58.6 dBc/Hz @ 1-Hz offset, −116.2 dBc/Hz @ 1-kHz offset, and −162.4 dBc/Hz @ 1-MHz offset. This phase noise curve of the free-running optomechanical comb fits the *f*^−2^ trend, suggesting that its noise is majorly determined by white noise. After stabilization, the SSB-PN is markedly suppressed and approaches −110.5 dBc/Hz @ 1-Hz offset, −156.5 dBc/Hz @ 1-kHz offset, and −163.1 dBc/Hz @ 1-MHz offset. The stability of this signal is already comparable to a rubidium atomic clock (10 MHz, Menlo System), as the gray dashed curve shows. In Fig. 4E, we display the Allan deviations, which are measured by a frequency counter during 200 s. In free running operation, because of the natural entropy increase, the Allan deviation of the mode-locked optomechanical oscillation grows up with gating time, from 7.8 × 10^−9^ @ 0.1 s to a max number of 2.2 × 10^−7^ @ 98 s. After stabilization, its Allan deviation approaches 2.6 × 10^−10^ @ 0.1 s and reaches 3 × 10^−12^ @ 20 s. Here, we also show that the Allan deviation of the stabilized optomechanical comb is comparable to a rubidium atomic clock as a reference. Above results verify that the graphene-assisted optomechanical phase locking could be pretty stable after frequency synchronization–based stabilization. To our knowledge, such a stable optomechanical oscillation has never been reported before, and its performance may indicate appealing opportunities for high-precision applications ranging from communication to sensing. More details are shown in note S3.

## DISCUSSION

In summary, we report the physics and performance of optomechanical comb formation in a graphene-assisted silica microresonator. The stable optomechanical pulses result from photon-phonon-electron interactions during the optical and mechanical oscillations. First, optomechanical oscillations are excited by the radiation pressure force of intracavity photons; then mechanical waves modulate the optical wave in turn, generating intensity modulation in optics; and last, the saturable absorption–based energy conversion in graphene locks this modulation, forming the co-resonating of the optical and acoustic standing waves. In the whole procedure, optomechanical gain and loss are balanced. By exploring the mode locking evolution, we also reveal that such a locking effect is deterministic, which definitely appears when the intracavity optical power is proper. The phase-locked spectrum containing 7.6 MHz and its harmonics demonstrates a uniform quasi-sech^2^ shape spectrally and delivers stable nanosecond pulses temporally. This suggests a potential to stabilize its repetition rate via frequency division technology and demonstrates a phase noise down to −110.5 dBc/Hz @ 1-Hz offset and an Allan deviation down to 3 × 10^−12^ @ 20 s. In addition, we have determined that the formation of mode-locked optomechanical combs primarily relies on the efficiency of photon-phonon coupling and the depth of saturable absorption. This indicates that such mode locking operations could be feasible in various microcavity architectures, including microdisk, toroid, and on-chip microrings. Furthermore, by optimizing both the optical and mechanical *Q* factors, the quality of an optomechanical comb could be significantly enhanced (*54*). The capability to localize phonons by graphene-based optoelectronic locking in a micro/nanogeometry bridges optomechanics and two-dimensional material optoelectronics and opens up a new avenue for cavity optomechanical applications, such as radio frequency clock, high-precision sensing, and miniature gyroscopy.

## MATERIALS AND METHODS

### Theoretical analysis of optomechanical mode locking

For describing optomechanical mode locking, we use optimized Heisenberg equations incorporating saturable absorption induced by graphene

$$\frac{\mathrm{da}}{\mathrm{dt}}=\left[i\left(\Delta +\mathrm{Gx}\right)-\frac{\kappa }{2}-\frac{\alpha }{2T_{r}}\right]a+\sqrt{\kappa_{\text{ex}}}s_{\text{in}}$$ (1)

$$\frac{d^{2}x}{{\mathrm{dt}}^{2}}+\kappa_{m}\frac{\mathrm{dx}}{\mathrm{dt}}+{\Omega_{m}}^{2}x=\frac{\hbar G}{m}{|\;a\;|}^{2}$$ (2)

In this equation, *a* is the normalized amplitude of the optical field, κ = κ_ex_ + κ_int_ is the total optical dissipation coefficient, κ_int_ is the intrinsic optical loss, κ_m_ is the mechanical dissipation coefficient, *m* is the effective mass of the oscillator, and *s*_in_ is the input photon flux. *G* = −*d*ω_0_/*dx* is the optomechanical coupling coefficient, and Δ = ω_p_ − ω_0_ is pump detuning. Besides, *T*_r_ is the round-trip time of the cavity, and α is the absorption coefficient, which is determined by *a*. Specifically, α = α_S_/[1 + *|a|*^2^/*P*_s_] + α_NS_. In this equation, α_S_ is the saturable absorption component, defining the modulation depth of our graphene saturable absorber, and α_NS_ is the nonsaturable absorption component, representing the residual absorbance when it is fully saturated. *P*_s_ is the saturation power, which indicates the value when absorption falls to half of its initial value. By using the split-step method, one can solve the stable solution both spectrally and temporally. More details are shown in note S1.

### Fabrication of the graphene-based microsphere

First, a silica microsphere located at the end of a commercial optical fiber (Corning SMF-28) is prepared by using the arc discharge technique in a programmable fiber fusion splicer (FITEL S178). By controlling the discharge power, discharge position, and duration, we can control the diameter of the microspheres with a scale error less than 1 μm. In this work, for optimizing the optical *Q* factor, we mainly use a fixed diameter *D* = 690 μm. Referring the group refractive index of silica *n*_g_ = 1.454 and the FSR = *c*/(*n*_g_π*D*), the optical FSR of the cavity is ≈95.2 GHz. Then, we prepare the high-quality crystalline single-layer graphene via polydimethylsiloxane-based mechanical exfoliation. Last, by using the dry-transfer technique, we deposit the graphene layer on the surface of the microsphere at a proper location. During our operation, by increasing the temperature on polydimethylsiloxane from room temperature to 60°C, graphene would be transferred onto the silica microsphere at the location we designed. We used in situ Raman spectroscopy characterizing the quality of the graphene on the microsphere, while we measured transmission and optomechanical *Q* factors of the hybrid microcavity. More details are shown in note S2.

### Experimental details of optomechanical comb generation

We use a tunable continuous-wave laser diode as the pump and fix its output power at 100 mW. To monitor the mechanical oscillation in radio frequency, we use another tunable laser as the reference. When tuning the pumping frequency, the frequency of the reference automatically follows it, controlled by an electronic synchronizer. During tuning, the frequency difference between the pump and the reference is always maintained at 2 GHz. The polarization controller is used to fix the pumping polarization to the transverse magnetic direction for selecting out the high-*Q* modes in our microsphere resonator, which contains hundreds of mode families. The graphene-based optomechanical microcavity is stabilized by a temperature electrical cooler. Output light signals from the optomechanical microcavity and reference laser are together collected by a fast photodetector. Their heterodyne beat note converts holographic optomechanical information from the optical band (≈194 THz) down to the radio frequency band (gigahertz level), facilitating direct measurement by an oscilloscope and electrical spectrometer in high resolution. More details are shown in note S3.

## Supplementary Materials

### This PDF file includes:

Supplementary Text Figs. S1 to S14 References
