# Supplementary material for: Mode-locked optomechanical frequency combs in a graphene-silica microresonator
Source: Sci Adv. 2025 Oct 22;11(43):eady1279. doi: 10.1126/sciadv.ady1279 (PMC12542935; doi:10.1126/sciadv.ady1279)
Supplement: Supplementary file 1 — Supplementary Text Figs. S1 to S14 References [file sciadv.ady1279_sm.pdf]

Science Advances
AAAS

Supplementary Materials for

Mode-locked optomechanical frequency combs in a graphene-silica microresonator

Hao Zhang *et al.*

Corresponding author: Bai-Cheng Yao, yaobaicheng@uestc.edu.cn; Qi-Fan Yang, leonardoyoung@pku.edu.cn; Yun-Feng Xiao, yfxiao@pku.edu.cn

Sci. Adv. **11**, eady1279 (2025)
DOI: 10.1126/sciadv.ady1279

This PDF file includes:

Supplementary Text
Figs. S1 to S14
References

Supplementary Text

Note S1. Theoretical analysis and numerical simulations.

S1.1 Simulation of the mechanical modes in a silica microsphere.

Due to the influence of the optomechanical backaction, the optical mode driven by the pump laser can excite numerous mechanical modes within the cavity. For instance, in a spherical silica microcavity (refractive index 1.46) with a diameter of 610μm (corresponding to the device in the maintext), which is fixed by a pillar at the bottom. More than one mechanical mode can be excited in the microsphere cavity, by comparing the calculated mechanical modes with those observed in experiments, we can identify a mode that closely match the experimental data in terms of eigenfrequency. Leveraging the finite element method in a commercial software COMSOL Multiphysics, **Fig. S1A** to **S1C** display the surface deformation and the normalized stress distribution in the graphene assisted cavity for the first three mechanical modes, where graphene flake is also considered when constructing the geometry of cavity in the software. This simulation can also show the calculated eigen-frequencies of the mechanical modes excited by the optical field in the cavity. In simulation, eigen-frequency (f_ε) is approximately proportional to the mode order, f_ε of the first, second and third order mechanical mode is 7.563 MHz, 15.025 MHz and 22.671 MHz, respectively. In our experiment, the usually observed mechanical eigen frequency is about 7.6 MHz. Therefore, for simplicity, we assume only the first order mechanical mode is excited during comb generation, avoiding complicated discussion with multiple mechanical modes involved (*55*).

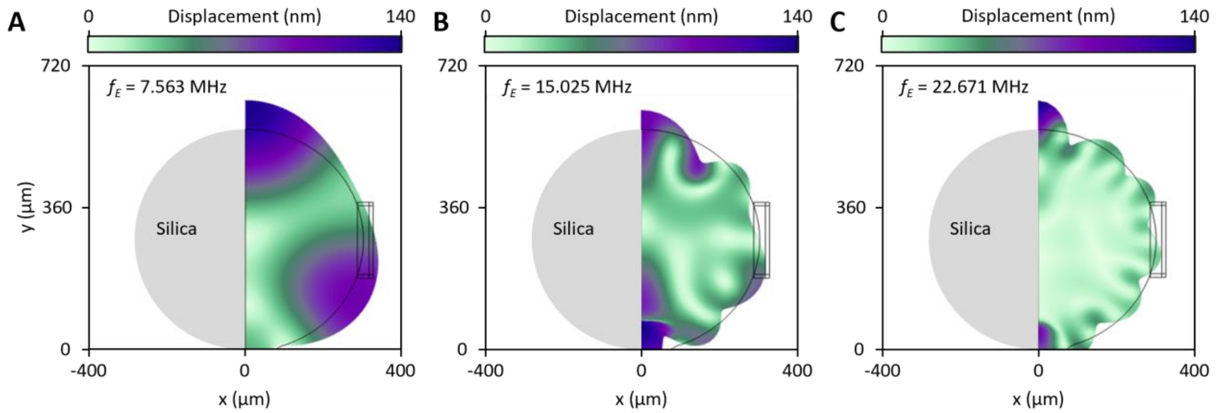

Fig. S1. Numerically simulated mechanical oscillations in the optomechanical process. The mechanical modes exhibit patterns symmetric about the pillar, and thus, we display the cross-section of the cavity in these figures. The semicircular lines represent the static surface of the microcavity without mechanical oscillations, while the colored areas correspond to the cavity's geometry during oscillation. Regions with higher stress are depicted with darker colors. In details, panels (**A**) **-** (**C**) illustrate the mechanical oscillations from the fundamental mode (1st), the second order mode and the third mode, respectively.

S1.2 Theoretical analysis of the optomechanical mode locking.

The Hamiltonian of an optomechanical system can be described in the frame rotating at pump frequency ω_p, which can be written as (*2*):

$$\hat{H}=\hat{H}_{free}+\hat{H}_{int}+\hat{H}_{drive}=-\hbar\Delta\hat{a}^{\dagger}\hat{a}+\hbar\Omega_m\hat{b}^{\dagger}\hat{b}-\hbar g_0\hat{a}^{\dagger}\hat{a}(\hat{b}^{\dagger}+\hat{b})+i\hbar\sqrt{\kappa_{ex}}s_{in} \tag{S1}$$

The three terms constitute total Hamiltonian representing uncoupled optical and mechanical modes, optomechanical interaction and pump driving, respectively. $\Delta=\omega_p-\omega_0$ is pump detuning, with ω_0 representing angular frequency of the nearest resonance from pump. $\hat{a}$ ($\hat{b}$) is photon (phonon) annihilation operator, and $\hat{a}^{\dagger}\hat{a}$ ($\hat{b}^{\dagger}\hat{b}$) equal to the number of photons (phonons) in the cavity. Ω_m is the angular oscillation frequency of mechanical mode. $g_0=Gx_{ZPF}$, among which $G = -d\omega_0/dx$ is the optomechanical coupling coefficient, indicating resonance frequency drift due to change of oscillator displacement x, and x_{ZPF} is zero-point fluctuation. In the last term, κ_{ex} is coupling coefficient and s_{in} is input photon flux. Based on Hamiltonian, dynamical equations can be deduced, which are classical Heisenberg equations of motion (*20*):

$$\frac{da}{dt}=[i(\Delta+Gx)-\frac{\kappa}{2}]a+\sqrt{\kappa_{ex}}s_{in} \tag{S2}$$

$$\frac{d^2x}{dt^2}+\kappa_m\frac{dx}{dt}+\Omega_m{}^2x=\frac{\hbar G}{m}|a|^2 \tag{S3}$$

Here, the two equations describe the evolution of optical field and mechanical displacement, respectively. They are coupled with each other. In the two equations, a is normalized amplitude of optical field, $\kappa=\kappa_{ex}+\kappa_{int}$ is the total optical dissipation coefficient, κ_{int} is intrinsic optical loss, κ_m is mechanical dissipation coefficient, and m is the effective mass of oscillator. To gain more insight, Eq (S2) can be broken down into two equations

$$\frac{dx}{dt}=\frac{p}{m} \tag{S4}$$

$$\frac{dp}{dt}=-\kappa_m p-m\Omega_m{}^2x+F_{rad} \tag{S5}$$

Where $p = mv$ is momentum of oscillator, $F_{rad} = \hbar G|a|^2$ is the optically-induced radiation pressure force acting on the boundary of cavity. On the right-hand side of Eq (S5), the three terms stand for momentum loss during oscillation, simple harmonic property of the oscillator ($d^2x/dt^2 = -\Omega_m{}^2x$ is satisfied, which means its acceleration is proportional to displacement, with opposite direction, driving the oscillator to move back and forth), and the holds of Newton's second law, respectively.

Since the period of the mechanical oscillator is much longer than photon lifetime, we can assume that the optical field is varying synchronously with mechanical displacement, without taking time to reach steady state. Under this hypothesis, the motion of mechanical oscillator can be regarded as vibrating sinusoidally around its equilibrium position (*56*), that is

$$x(t)=A\sin\Omega_m t \tag{S6}$$

A is oscillation amplitude. The intracavity optical field can be expressed as

$$a(t)=\sqrt{\kappa_{ex}}s_{in}e^{i\varphi(t)}\sum_n i^n\alpha_n e^{in\Omega_m t} \tag{S7}$$

Where ϕ(t)=-βcos($\Omega_m t$) is the global phase, and $\beta=GA/\Omega_m$ is normalized oscillation amplitude. The expression of α_n is

$$\alpha_n=\frac{J_n(\beta)}{\kappa/2+i(n\Omega_m-\Delta)} \tag{S8}$$

J_n is the nth order Bessel function of the first kind.

With these solutions, the mechanical gain spectrum can be calculated in the plane spanned by normalized detuning and oscillation amplitude, with the gain defined by the ratio of average power of radiation pressure force and friction force, in one period.

$$P_{rad} = \hbar G \left\langle |a|^2 \dot{x} \right\rangle \tag{S9}$$

$$P_{fric} = m_{eff} \kappa_m \left\langle \dot{x}^2 \right\rangle \tag{S10}$$

With experimentally measured parameters of our pristine microsphere without graphene (FSR = 90 GHz, κ = 2π×2 MHz, κ_{ex} = κ/2, pump power = 256 mW, Ω_m = 7.6 MHz, κ_m = 6.38 kHz, G = 4×10^{18} rad/s/m, m = 2.48×10^{-7} kg), the gain spectrum is calculated and shown in **Fig.S2A**.

By performing the same analysis, while replacing original expression for α_n by

$$\alpha_n = \frac{J_n(\beta)}{\frac{\kappa}{2} + \frac{\alpha_s}{2T_r(1+\frac{\hbar\omega_p}{T_r P_s}|a_0|^2)} + i(n\Omega_m - \Delta)} \tag{S11}$$

Here a_0 is the optical field at previous moment, in other words, if current time is t, then $a_0 = a(t-\Delta t)$, Δt is the smallest time unit. Taking $P_s = 3.2\times10^{-2}$ W, α_S=0.1, and α_{NS} is assumed to be 0 for simplicity, the gain spectrum for graphene-assisted microsphere is calculated, as shown in **Fig.S2B**.

The distribution of gain in the diagram is very likely to the one shown in **Fig.S2A**, just a slight broadening of each gain area. In **Fig. S2C**, we show their difference. By subtracting the gain spectrum in **Fig. S2B** from that in **Fig. S2A**, we make it easier to decipher the disparity. According to Fig.S2C, it can be found at the regions with relatively stronger intensity, their difference is not evident. Within these areas, when the amplitude is relatively large, the case with graphene exhibits a higher gain, whereas at smaller amplitudes, the case without graphene shows a higher gain.

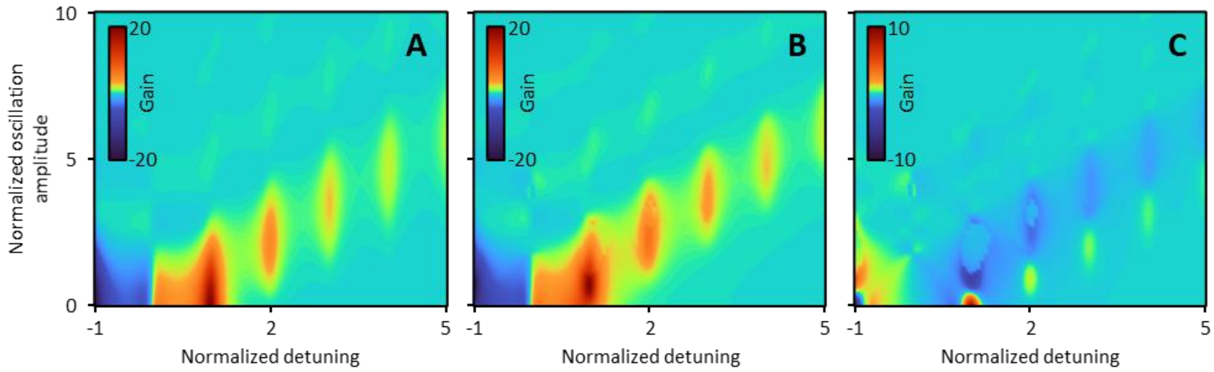

Fig. S2. Parametric space. Mechanical gain spectrum in a silica microsphere: **(A)** without graphene deposition, **(B)** with graphene deposition, and **(C)** their difference.

This indicates that the saturable adsorption does not have pronounced influence on the dynamics of mechanical gain. Therefore, we can understand the role of saturable adsorption in mode locking as energy 'redistribution' or 'reshape' of the temporal and spectral profile, which ensues after harmonics are generated through optomechanical backaction, as long as the average intracavity power stays at a proper value compared with saturation power, enabling optical components with different power to experience discriminatory loss. This also explains why mode locking is not observed when pump is on resonance, instead, it happens when detuning becomes

large enough in the scanning process from zero detuning to eigenfrequency of mechanical mode, shown in **Fig.2** in maintext. In this process, with increased detuning, on one hand, more mechanical gain is supplied, so that more harmonics are generated and optomechanical modulation on optical field is stronger, on the other hand, the intracavity pump power, as the main constituent of intracavity power, drops with increased detuning, closer to saturation power, so saturable adsorption can play a more evident role on shaping the optomechanically modulated fields. These two processes work in synergy, lead to mode locking.

From the gain spectrum, it can be observed that mechanical mode usually experiences gain when pump laser is blue-detuned, which is also verified by the scattering picture (*2*) . Moreover, under a fixed detuning, the system tends to operate at the regime with higher gain, so that the mechanical oscillation amplitude is amplified with increased detuning (*45*). When detuning is equal to the eigenfrequency of mechanical mode, i.e., normalized detuning=1, largest gain appears. This can be easily understood by an analogy that when the rhythm of external driving force coincides with inherent oscillation frequency of an oscillator, the oscillation can be maximized. Except when it equals to eigenfrequency, the gain also situates around the integer multiples of this detuning, with decaying trend.

Based on parameters above, we solve equation (S2), (S4), and (S5) numerically by using the slip-step method. The spectra and output temporal profiles under different detunings are shown in **Fig. S3**, the output field is calculated by input-output relation:

$$s_{out} = s_{in} - \sqrt{\kappa_{ex}}a \tag{S12}$$

In this simulation, we find that during the pump frequency blue detuning, energy transfers from photons to phonons, until the pump frequency enters very deep blue-detuned region. With pump frequency increasing, optomechanical oscillation becomes stronger, which can be told from the width of spectrum, because spectrum width can be approximated by $2\beta\Omega_{\mathrm{m}}$. In **Fig. S3A** and **S3B**, we demonstrate the calculated evolution process. Specifically, **Fig. S3A** shows the spectral evolution while **Fig. S3B** displays the temporal evolution. One can see that in a silica microcavity without graphene deposition, optomechanical oscillation can be excited but it cannot enter mode locking state. Typically, in frequency domain, it cannot form a wide and symmetrical comb-like spectrum, and in time domain, it cannot deliver sharp pulses.

More in details, **Fig. S3C** to **S3F** demonstrate several frames during the evolution. In **Fig. S3C**, we show the simulated spectra and temporal traces when $\Delta = 0$. Here we see obvious optomechanical oscillation, the oscillating state demonstrates < 10 strong oscillating lines. Therefore, in time domain, it outputs quasi sinusoidal wave. With increasing detuning, the spectrum broadens and temporal trace alters. When $\Delta = \Omega_m$, the spectrum is widest, since it feels the largest gain at this detuning, which coincides with gain spectrum in **Fig.S2**.

When it comes to graphene-assisted microsphere, saturable adsorption should be taken into account, which can be described by (*57*):

$$\alpha = \frac{\alpha_S}{1+\frac{|E|^2}{P_s}} + \alpha_{NS} \tag{S13}$$

Here α(E) is absorption coefficient, α_S is saturable absorption component, defining modulation depth of our graphene saturable absorber, and α_{NS} is nonsaturable absorption component, representing the residual absorbance when it is fully saturated. E satisfies $|E|^2T_{\mathrm{r}} = \hbar\omega_p|a|^2$, T_{r} is cavity roundtrip time. P_s is the saturation power, which indicates the value when absorption falls

to half of its initial value. Casting Eq (S13) into Eq (S2), we arrive at the optical field equation that incorporates saturable absorption brought by graphene,

$$\frac{da}{dt} = [i(\Delta + Gx) - \frac{\kappa}{2} - \frac{\alpha}{2Tr}]a + \sqrt{\kappa_{ex}} s_{in} \tag{S14}$$

Compared to conventional optomechanical oscillation in a pristine silica microsphere, graphene introduces an additional modulation in optics. In time domain, this helps to suppress low-intensity parts while retaining high-intensity parts. As a result, it offers a way to form stable pulses.

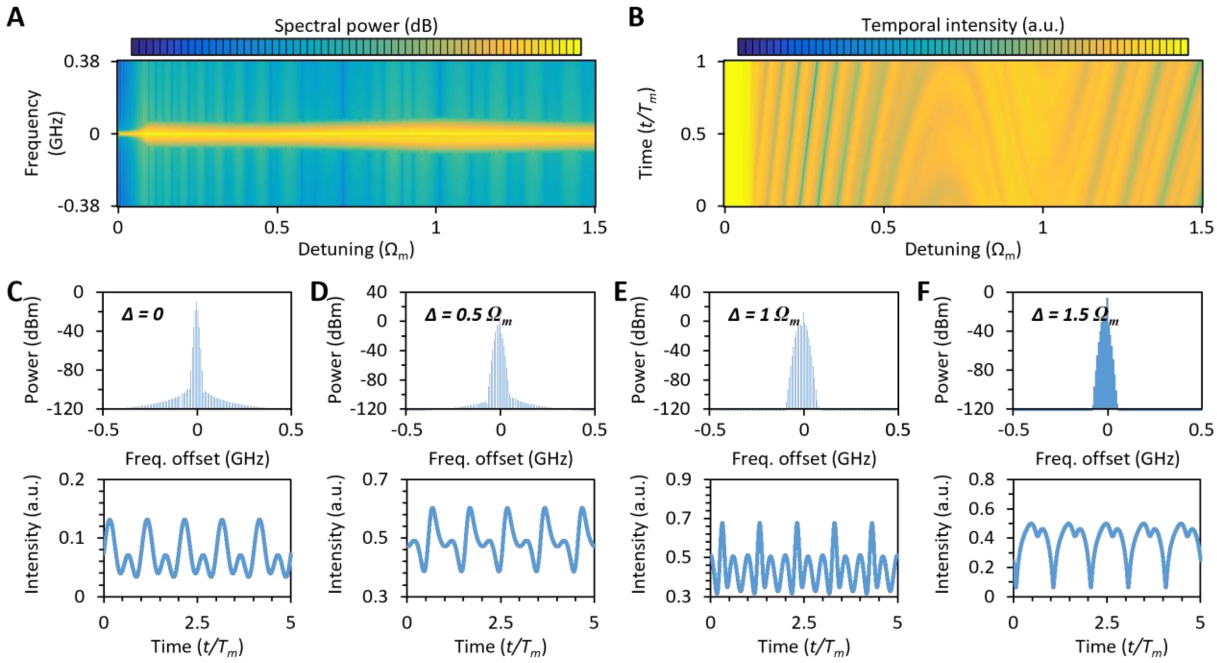

Fig. S3. Optomechanical oscillation in a silica microsphere without graphene deposition. (A) & (B) Spectral and temporal evolution when increasing the pump detuning from 0 to 1.5Ω_m. **(C) - (F)** Specific frames during the evolution. Top panels: spectra. Bottom panels: temporal traces. Here T_m suggests a period of oscillation.

In **Fig. S4**, we calculate the case that optomechanical oscillation is generated in a graphene based microsphere, with the same parameters as above. First, **Fig. S4A** and **S4B** shows the spectral and temporal evolution. It is notable that the pulse width is narrowing with increased detuning, agreeing with the discussion above, where enhanced mechanical gain and more marked saturable adsorption enable spectrum broadening. With increasing the detuning from 0 to 0.9Ω_m, one can observe that the optomechanical oscillation traverses from sinusoidal state (i), transition state (ii) to mode locking state (iii). Before entering the single pulse state, multiple pulse may exist in a single roundtrip, this is similar to the formation of optical mode locking. In **Fig. S4C**, we show the calculated intracavity absorption ratios, for the state (i) to (iii). With increasing the detuning, intracavity power decreases, this boosts the maximum absorption gradually, from < 10^{-7} to 2%. In state (i), all the power in one mechanical cycle is far beyond saturation power, and the absorbance of graphene is so small that saturable absorption hardly plays a role in field evolution. The modulation in absorption leads to pulse narrowing, finally forms stable pulses, as **Fig. S4D** shows. In **Fig. S4E**, we plot the simulated spectra of state (i), (ii) and (iii). It is clear that in state (ii), the

Stokes sidebands are relatively weak compared to Anti-Stokes sidebands, which is different from general case. when approaching the mode locking state, its spectral is in quasi sech^2 shape.

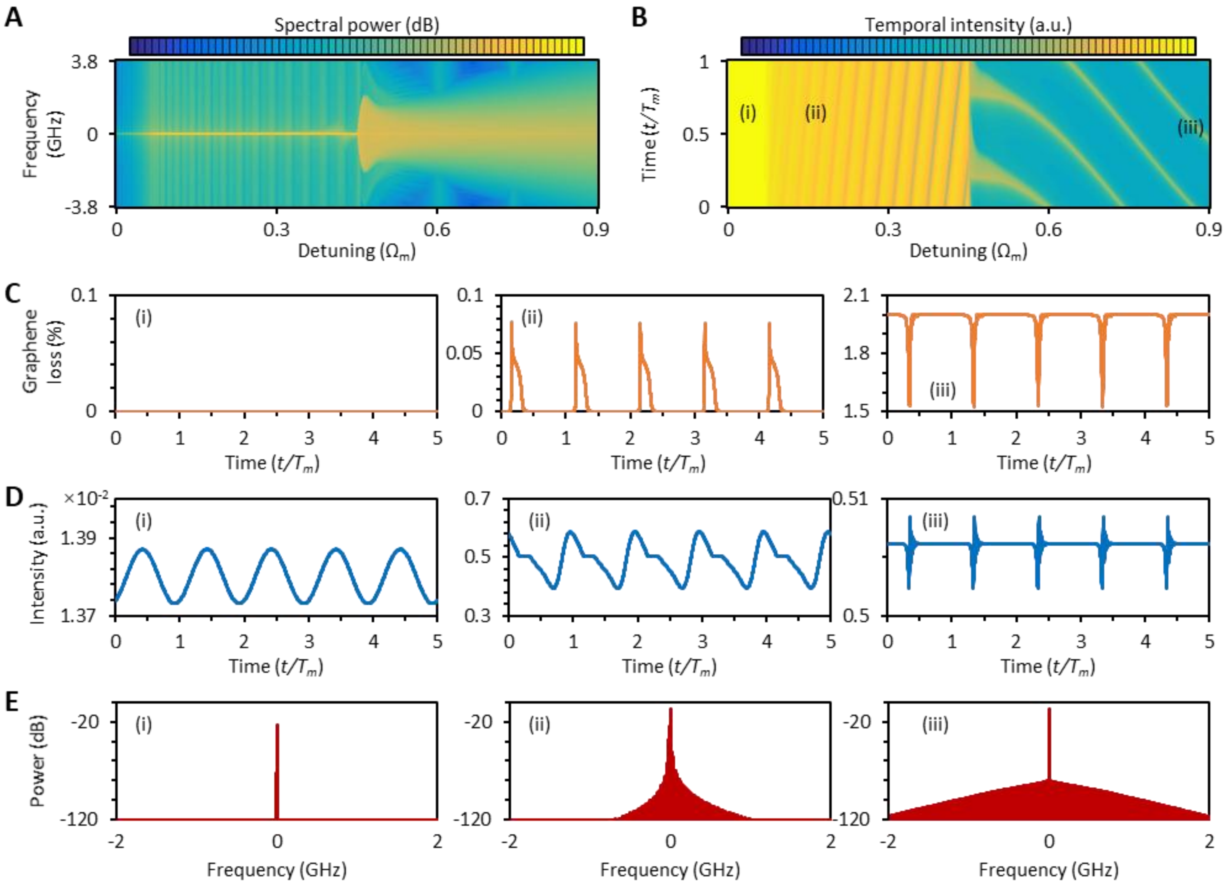

Fig. S4. Optomechanical oscillation in a graphene assisted silica microsphere. (A) & (B) Spectral and temporal evolution when increasing the pump detuning from 0 to 0.9Ω_m. **(C)** Graphene loss rate at specific frames during the evolution. **(D)** Temporal profiles of output field. **(E)** Spectra of intracavity field.

Now, we delve deeper into the influence of graphene-induced absorption. As previously mentioned, it is crucial to ensure a sufficiently high Q factor for the microcavity to stimulate optomechanical oscillation under an appropriate pump power. Simultaneously, graphene's saturable absorption is vital for forming a mode-locking state, requiring adequate modulation depth. This presents a trade-off, as illustrated schematically in **Fig. S5A**. In **Fig. S5B**, we conduct parametric simulations to identify conditions under which a microresonator can achieve a mode-locking state with a constant pump power of 100 mW. The red region delineates the parametric space conducive to mode-locking operation. When the optical Q factor falls below 6.8×10^7, optomechanical oscillation is not efficiently excited due to insufficient photon-phonon energy transfer (yellow region). Moreover, if the modulation depth from graphene saturable absorption is less than 0.063% per roundtrip, mode locking fails to occur, as it does not satisfy the pulse formation condition (light blue region). As for the sand color region, both the quality factor and modulation depth cannot support mode locking. In this figure, the white dot represents the

microcavity sample discussed in the main text, the orange box denotes sample #5 in **Fig. S8**, and the green triangle indicates sample #7 in **Fig. S8**.

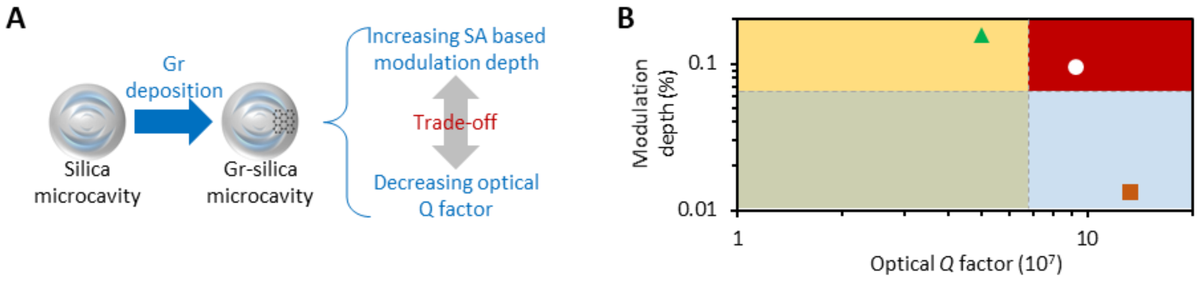

Fig. S5. Trade-off induced by graphene deposition. (A) Graphene deposition enables saturable absorption but inevitably decreases the optical *Q* factor. **(B)** For a fixed 100 mW pump power, parametric space shows that optomechanical mode locking tends to occur when both the optical *Q* factor and graphene-based modulation depth are high. Red: conducive for mode locking, yellow: insufficient quality factor, light blue: insufficient modulation depth, sand: insufficient quality factor and modulation depth.

S1.3 Extended discussion about the influences from other nonlinearities.

Now we consider if other optical nonlinear processes affect the comb formation. Both Kerr and Raman effect are widely-existing cubic nonlinearities in microresonators, while cavity optomechanics can still be regarded as cubic nonlinearities. Interestingly, optomechanical effect and cascaded four wave mixing share the similar rate equation, while in the temporal response function it exhibits delayed property that is analogous to Raman effect (*20*). Therefore, there would exists energy competition between optomechanical and the other two effects that you mentioned. For four wave mixing, its pump power threshold can be described by (*58*):

$$P_{th} = \frac{\pi n_0 \omega_0 S_{eff}}{4\eta n_2} \frac{1}{D_1 Q^2} \qquad \text{(S15)}$$

Here n_0 is refractive index, ω_0 is center angular frequency, S_{eff} is effective mode area, η is coupling strength, n_2 is Kerr nonlinear refractive index and Q is optical quality factor. According to parameters of our microsphere, taking $n_0 = 1.44$, $\omega_0 = 2\pi\times193.42\times10^{12}$ rad/s, $S_{\mathrm{eff}} = 80\ \mu\mathrm{m}^2$, $\eta = 0.5$ (critical coupling is assumed), $n_2 = 2.6\times10^{-20}$ m^2/W (for silica), $D_1 = 2\pi\times95.2\times10^9$ rad/s, and $Q = 9\times10^7$. The calculated pump power threshold for four wave mixing is equal to 1.7 mW. In our experiment, pump power maintains 200 mW, which has far exceeded threshold for four wave mixing, but when optomechanical comb generated, there is no four wave mixing sideband observed.

This can be explained that under this pump power, optomechanical effect is the dominant effect, once it is excited, four wave mixing is suppressed because pump energy is despoiled by the former. To validate this, we consider equations of motion of graphene-assisted mode-locked optomechanical comb in both situations. With considering four-wave-mixing:

$$\frac{da}{dt} = [i(\Delta + Gx) - \frac{\kappa}{2} - \frac{\alpha}{2T_r} + ig|a|^2]a + \sqrt{\kappa_{ex}} s_{in} \qquad \text{(S16)}$$

$$\frac{d^2x}{dt^2} + \kappa_m \frac{dx}{dt} \Omega_m{}^2 x = \frac{\hbar G}{m}|a|^2 \qquad \text{(S17)}$$

Here g is Kerr nonlinear coefficient. Based on above equations, along with equation (S13) and (S14), we show the simulated spectra in **Fig. S6A and S6B**. There is no obvious difference between the two spectra, thus four wave mixing indeed plays an insignificant role in optomechanical comb generation.

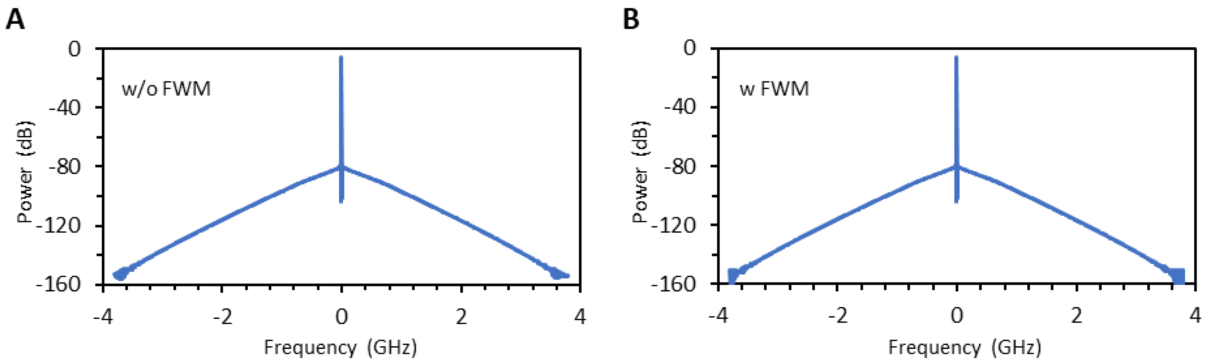

Fig. S6. Simulated spectra of mode-locked optomechanical comb with and without considering four wave mixing. (A) Without four wave mixing term. (B) With four wave mixing term.

Similarly, Raman scattering could be treated in the same way. Under our pumping power (200 mW), optomechanical effect overwhelms Raman effect, making Raman lasing hard to happen as well. In addition, the Raman-induced soliton self-frequency shift (SSFS) that commonly appears in Kerr comb has not been observed here. The main reason for this might be that SSFS has strong dependence on peak power of pulses (*59*), and the peak power of a Kerr soliton pulse is on the order of hundreds of watts, while the intracavity peak power of our optomechanical pulse is only sub-watt. Thus, Raman scattering does not affect our comb generation process. In our previous work (*43*), the similar cavity configuration, i.e. graphene based microsphere, can generate Kerr-Raman soliton pairs under a 120 mW pump power. However, in that experiment, pump frequency was red-detuned. In that case, optomechanical oscillation was suppressed. Therefore, we conclude that when tuning the pump frequency into the deeply blue-detuned region of a resonance, due to energy competition, other nonlinearities such as four wave mixing and Raman scattering would be suppressed.

Note S2. Device fabrication and characterization.

Figure S7 shows the nanofabrication process of our graphene coated microsphere resonators. The silica microsphere is located at the end of a commercial optical fiber (Corning SMF-28). The microsphere samples are produced by using the arc discharge technique in a programmable fiber fusion splicer (FITEL S178). By controlling the discharge power, discharge position and duration, we can control the diameter of the microspheres with a scale error less than 1 μm. In this work, for optimizing the optical Q factor, we mainly use a fixed diameter D = 690 μm. Referring the group refractive index of silica n_g = 1.454 and the FSR = $c/n_g\pi D$, optical FSR of the cavity is ≈ 95.2 GHz. Then, we prepare the high-quality crystalline single layer graphene via PDMS based mechanical exfoliation (*29*). In this process, repeated exfoliation enables that we can get large monolayer graphene samples with hundreds of um^2 size. Then, by using the dry-transfer technique, we deposit the graphene layer on the surface of the microsphere at a proper location. During our operation, by increasing the temperature on PDMS from room temperature to 60 ^{o}C, graphene would be

transferred onto the silica microsphere at the location we designed. For best ensuring the light-graphene overlapping meanwhile avoiding potential risk of thermal burning, we deposit the graphene at 10° above the equatorial plane.

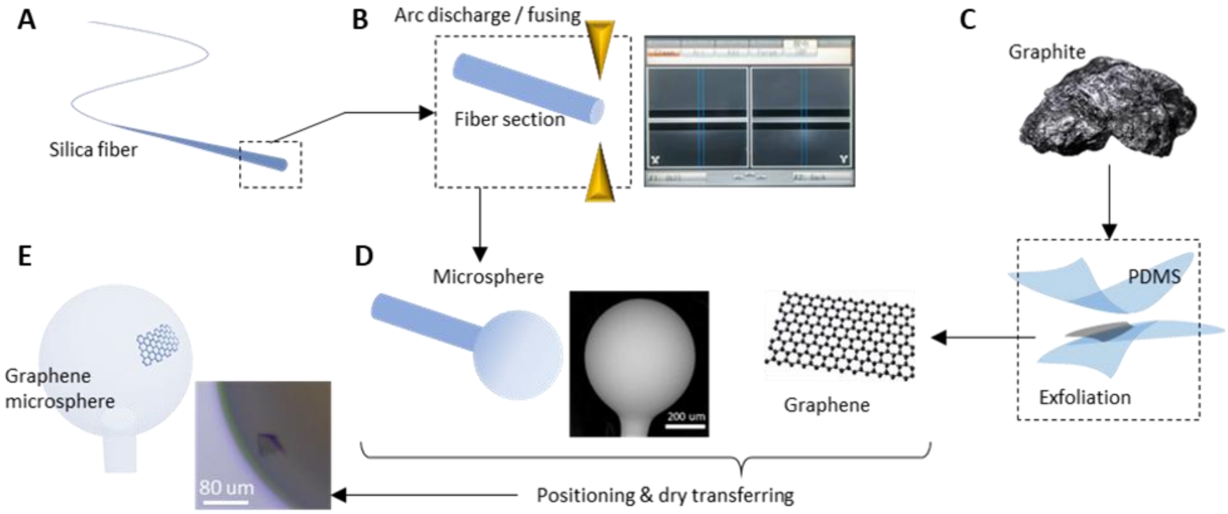

Fig. S7. Nano-fabrication of the graphene-microsphere device. (A) Raw material of the microcavity is silica fiber. **(B)** Arc discharge fuses the silica and form the microsphere. **(C)** Graphene exfoliation from natural graphite. (**D)** Combination of the graphene and the microsphere via dry transfer. **(E)** End product, a graphene based microsphere cavity, on which graphene is deposited at 10° above the equatorial plane.

We compare the passive transmissions of the microsphere cavity, before and after graphene deposition in **Fig. S8**. **Fig. S8A** plots the transmission spectra in a 100 GHz region, around 1550 nm optical band. Typically when its Fermi level < 0.2 eV, optical refractive index of graphene is about 3 around 1550 nm (*60*). Considering the cavity perimeter is 2.26 mm while the graphene's effective length is 40 μm, and the light-graphene overlapping is < 4%, the graphene induced FSR alteration is negligible. But the graphene deposition slightly breaks the spatial symmetry up and down the microsphere equator, it helps to suppress the transverse mode number. Specifically, by carefully keeping the polarization, in the microsphere cavity before graphene deposition, we can find 257 independent resonances in one single FSR, while in the microsphere cavity after graphene deposition, every FSR contains 126 independent resonances.

For instance, **Fig. S8B** plots more details in the 40-45 GHz band. We can count that before graphene deposition, there are 37 longitudinal modes, and after deposition, there are 19 longitudinal modes. This verifies that the graphene deposition induced loss and symmetry breaking suppress many high-order modes. In **Fig. S8C**, we also show a single resonance of the same mode before and after graphene deposition (@1550 nm – 40.336 GHz). Here graphene induced resonance linewidth broadening is clear, due to the absorption. Before graphene deposition, loaded Q factor is 1.4×10^8, after graphene deposition, the loaded Q factor is kept on 9×10^7. In **Fig. S8D**, we plot the coupling trace of this mode, here the grey dot and the red dot show the case before and after graphene deposition. Since the intracavity graphene decreases the intrinsic Q, the resonance is pushed to further under coupling region. In this measurement, we fix the fiber-cavity coupling efficiency $\kappa_{ext} \approx 5.06\times10^6$ s^{-1}, therefore the $Q_{ext} \approx 2.4\times10^8$. **Fig. S8E** showcases the measured optical Q factors of ten individual samples. Before graphene deposition, we can well ensure that

loaded Q factor of every microsphere cavity is higher than 10^8. Then, the graphene deposition reduces the Q factor due to intrinsic absorption. In different samples, there are slight differences in the size and depositing position of graphene, so after graphene attachment, the Q values of these samples range from 0.7 to 1.4×10^8. In experiment, we find that when using sample #5 and sample #8, we cannot obtain mode locked optomechanical comb, as the graphene induced modulation depth is too small; when using sample #7, when using a 100 mW pump, there are few optomechanically generated sidebands, failing to form a comb, not to mention mode locking, because Q factor of this sample (4.93×10^7) is relatively low.

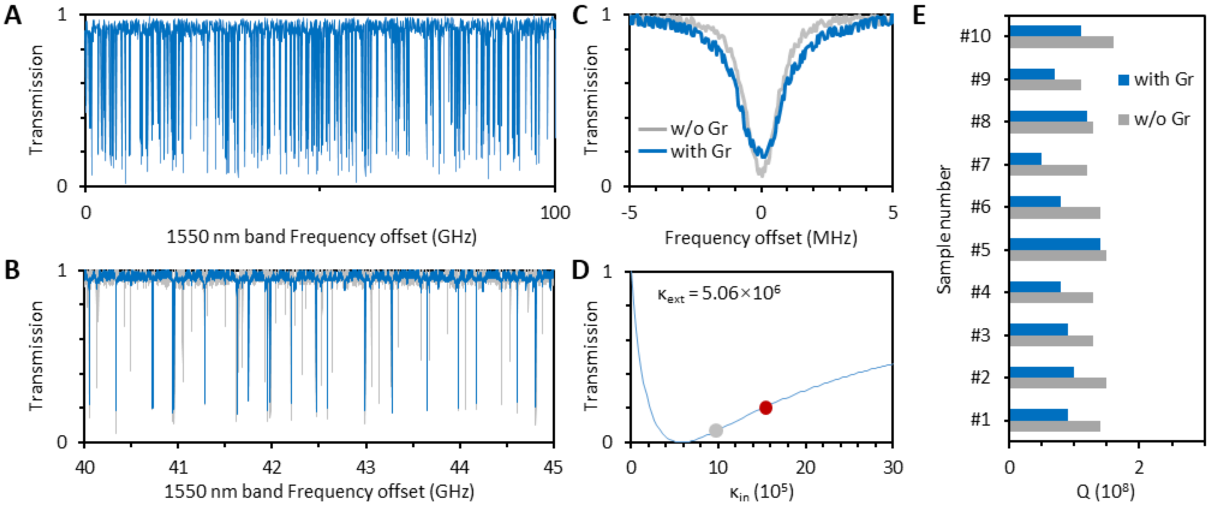

Fig. S8. Cavity transmission measurements. (A) Broadband transmission of a microsphere cavity before (grey) and after (blue) graphene deposition. **(B)** Zoomed-in traces in the panel **A**. Here we show a 5 GHz wide section. **(C)** Measured dip spectra, for a specific resonance, before and after graphene deposition. The resonance linewidth increases from 1.4 MHz to 2 MHz. **(D)** Coupling trace of this mode, before and after graphene deposition. **(E)** Q factors of ten microcavity samples, before and after graphene deposition.

Figure S9 characterizes the quality of the graphene sample on the silica microresonator. **Fig. S9A** shows the microscopic picture of the exfoliated graphene, with a size 55×45 μm^2. **Fig. S9B** maps the measured Raman spectrum of the graphene sample, by using a Raman analyzer (Renishaw InVia). We excite the graphene based Raman scattering by using a 514 nm continuous wave laser, with 0.15 mW optical power, far below the nonlinear threshold and avoid heating damage. Except for the tears, negligible D peak and intensity ratio of the G/2D peak reaching 60% suggests defectless quality of the single layer. Position of the G peak and the 2D peak are 1584 nm (linewidth < 22 cm^{-1}) and 2687 nm (linewidth < 30 cm^{-1}), suggesting the original Fermi level of the graphene sample on the silica microsphere is ≈ 0.2 eV, due to the natural p-doping. Besides measuring at one point, we map the in-situ Raman maps (20×20 μm^2) in **Fig. S9C**. Here we show intensities of the D, G and 2D peaks at different locations, the measured area is marked in **Fig. S9A**. Specifically, D peak intensity varies in 0 ~ 0.07 a.u., G peak intensity varies in 0.59 ~ 0.62 a.u., D peak intensity varies in 0.89 ~ 1 a.u. Experimental results verify that the graphene is uniform. At varied locations, the Raman spectra show good consistence.

Both interband and intraband saturable absorption in graphene were widely reported (*61*), this property determines the material dynamic loss. The saturation optical intensity, i.e. the optical

intensity required for a steady state to reduce the absorption to half of its unbleached value, can be used for estimating the nonlinear absorption (*62*):

$$a = \frac{a_s}{1 + I / I_s} + a_{NS} \tag{S15}$$

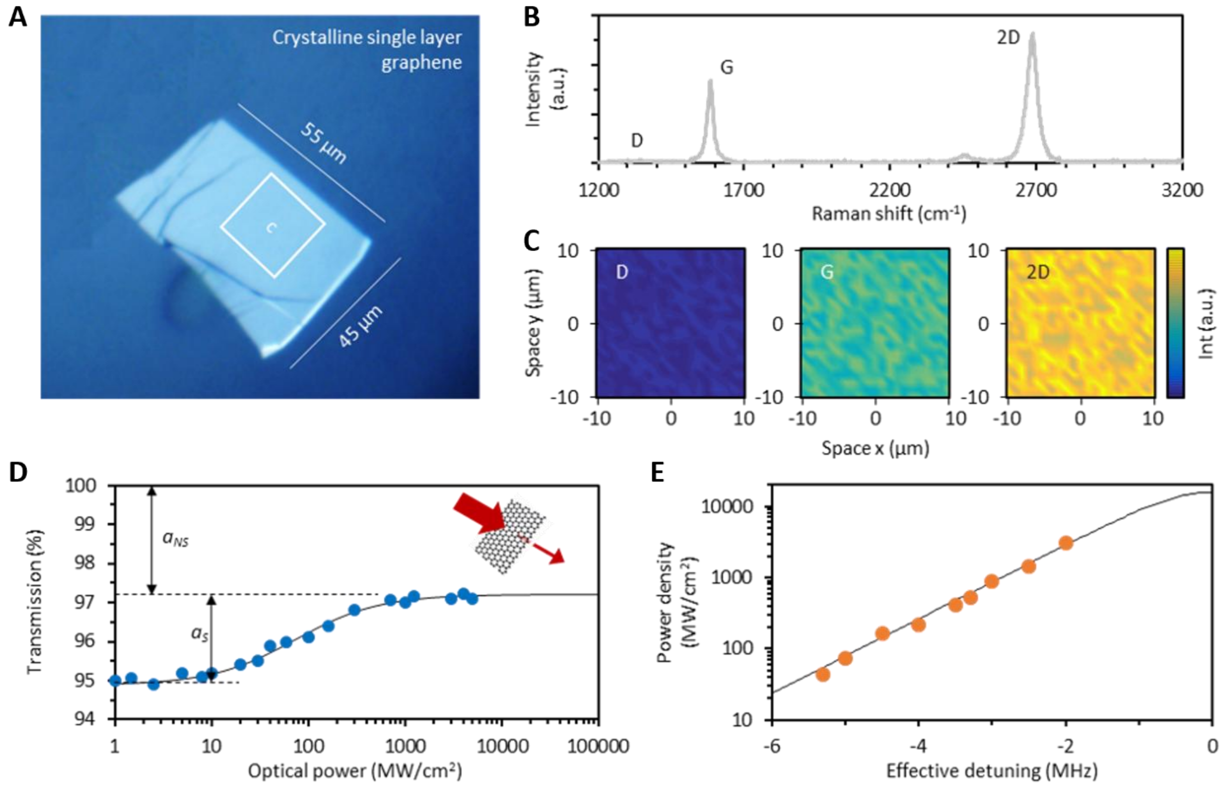

Fig. S9. Characterization of the graphene sample. (A) Microscopic picture of the graphene sample. **(B)** A measured Raman spectrum. **(C)** In-situ 2D maps show that the peak intensities of D, G, 2D peaks of the graphene is consistent, in a 20×20 μm^2 area. **(D)** Measured saturable absorption of the exfoliated graphene sample for intracavity deposition. **(E)** Estimated intracavity power density when tuning a 200 mW continuous wave laser into a resonance with a loaded Q = 9×10^7.

In above equation, a_S and a_{NS} are the saturable and nonsaturable absorption ratio, I_S is the saturation intensity. Defined by the fine structure constant $e^2/\hbar c$, the maximum a_S is 2.3% (*63*). In **Fig. S8D**, we measure the nonlinear transmission of our graphene sample on silica (before transferring the sample onto the microsphere). In this measurement we use a pulsed laser with peak power 2 kW, and the focused spot on graphene is 4 μm^2. Therefore maximum power density on graphene is 50 GW/cm^2. The measured results suggest that I_S = 74 MW/cm^2, and the a_{NS} ≈ 2.8%. **Fig. S8E** demonstrates the estimated intracavity power density when detuning the 200 mW pump laser into a resonance with Q factor 9×10^7. Referring the TM_{01} mode for instance (mode area ≈ 100μm^2), When the effective detuning reaches 3 MHz, the intracavity power can be calculated by

$$P_{cavity} = \frac{\kappa_{ex} P}{T_r(\Delta^2 + (\kappa / 2)^2} \tag{S16}$$

So, the power density is the ratio of intracavity power and effective mode area, calculated to be 341.32 MW/cm^2, sufficient for the absolute saturation of graphene.

Note S3. Extended measurements.

We present more optomechanical oscillation tests in **Fig. S10**. **Fig. S10A** shows the Optomechanical excitations at different pump powers. When the pump power is 0.2 mW, only three optomechanical modes are visible. As the power increases, the lower frequency optomechanical modes begin to lase, and more mechanical modes are observed. The zoomed-in optomechanical resonances are shown in **Fig. S10B**. We also measured the response frequencies of optomechanical modes of different orders and their corresponding measurement errors, as shown in **Fig. S10C**. It can be seen that there is a good linear relationship between the response frequencies of the mechanical modes of different orders, with the maximum measurement error not exceeding 2 kHz. In **Fig. S10D**, we further measured the mechanical Q factors of the modes with different orders. It can be observed that the mechanical Q factor generally decreases as the mode order number increases.

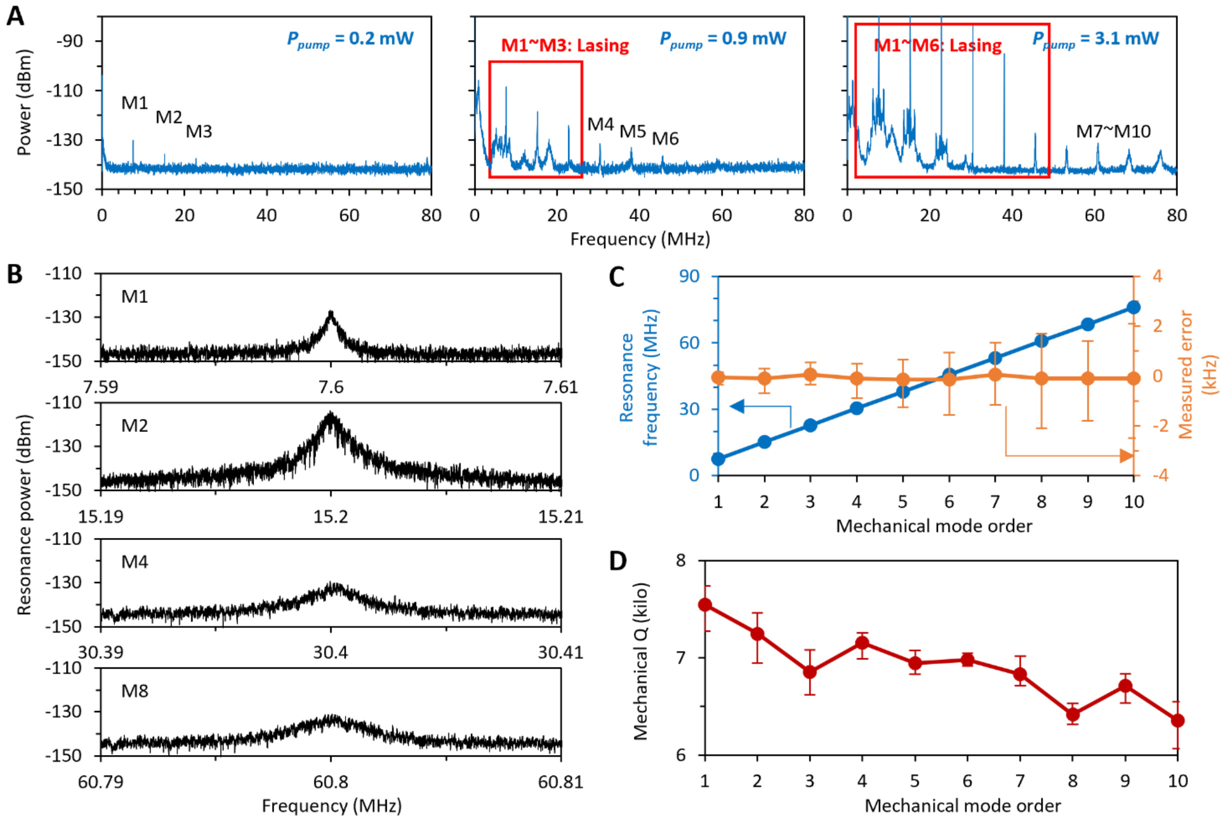

Fig. S10. Measured optomechanic modes under lasing threshold. (A) Optomechanic excitations under varied pumping powers. The optomechanic oscillation has an eigenfrequency 7.6 MHz, high optical power can excite harmonic resonances. **(B)** Measured optomechanic resonances. **(C)** For diverse mode order, we mark their response frequencies and measured errors. **(D)** Measured Q factors of the optomechanic resonances. M1 to M10 marks the mode orders, error bars show the uncertainties in repeated measurements.

For comparison, we measure the optomechanical oscillation in a silica microsphere without graphene deposition, as shown in **Fig. S11**. **Fig. S11A** is the microscopic picture of our silica microsphere, with the same diameter of ≈ 690 µm. Following the same measurement method as in **Fig. 2**, we set the pump laser wavelength near 1550.321 nm and tune it from the blue side into resonance, and then tune it back. In contrast to the results for the graphene-assistant microcavity, the evolution of the optomechanical oscillation in the microsphere without graphene deposition does not manifest mode-locking behavior, as shown in **Fig. S11B**. We choose three frames i-iii during the evolution and show their temporal and spectral profile in **Fig.11C**. At state i, it shows chaotic behavior because of high input power, then in state ii, its temporal profile becomes periodic, with asymmetric frequency spectrum similar to conventional optomechanical comb. After increasing detuning, its spectrum is broadened, remaining asymmetry, shown as state iii. The experiment is repeated for multiple pristine samples with similar parameters, and we obtain similar results without mode locking observed. This further demonstrates that graphene is indispensable in our mode-locking scheme.

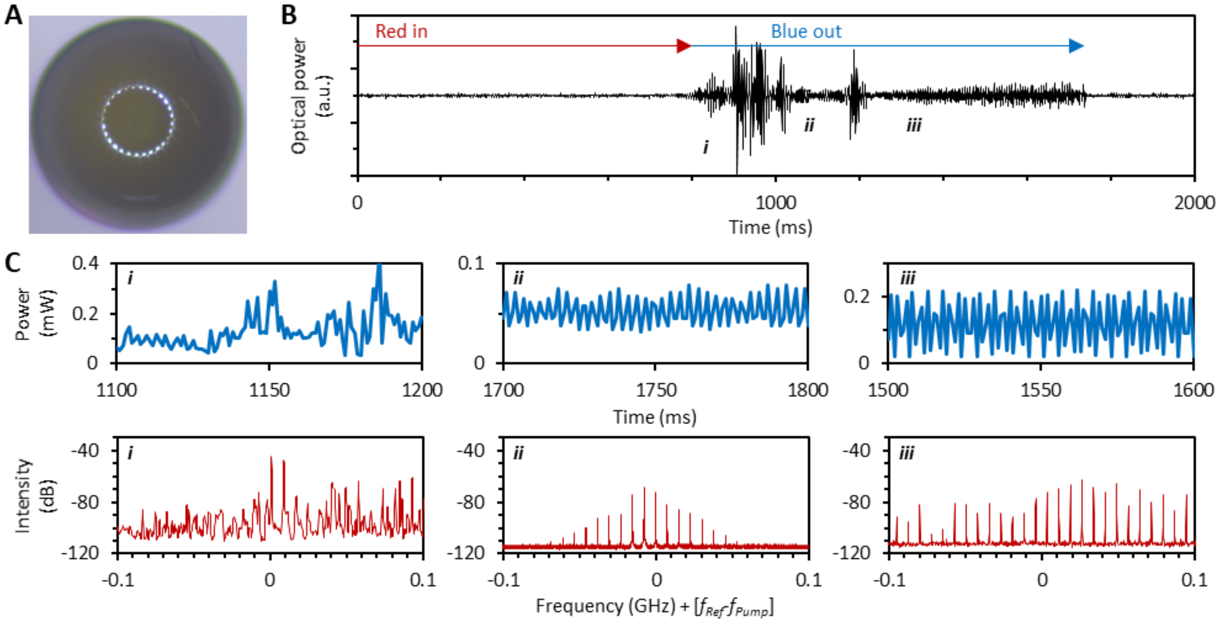

Fig. S11. Optomechanical oscillation in a silica microsphere without graphene deposition. (A) Picture of the silica microsphere, with the same diameter ≈ 690 µm. **(B)** Measured evolution trace when launching a pump laser into the resonance around 1550.321 nm. The optomechanical wave evolves from chaotic state (i), periodic state (ii), to transition state (iii). **(C)** Measured temporal traces (blue) and radio frequency spectra (red) of the 3 states, during the pump tuning process.

In **Fig. S12**, we show that without using an optical reference trigger, one can also test the optomechanical oscillation in a graphene assisted silica microcavity. Specifically, **Fig. S12A** and **S12B** display three typical states: (i) Transition state; (ii) Two-pulse locking state; (iii) Single pulse locking state. More in details, **Fig. S12A** demonstrates the pump laser detuning, from 1550.167 nm to 1550.148 nm. During this process, Δ increases by 2.37 GHz. Such a large decrement still keep the pump laser in an optical resonance, due to the thermal effect. In Fig. **S12B**, we show the directly measured spectra in a radio frequency spectrometer. Interval of the comb lines is 7.6 MHz. In state (i), phase of the comb lines are not coherent; in state (ii), we see lotus like envelop in the

single sideband spectrum, due to dual pulse interference; in state (iii), the spectral envelop is smooth, and in quasi sech^2 shape, suggesting stable single pulse mode locking. We note that optical mode in this measurement is different from the mode shown in the maintext, thus bandwidth of the mode locking spectrum here is smaller than the case in the maintext. In **Fig. S12C**, we show the zoomed in spectrum of state (iii) of **Fig. S12B**. Without using a reference laser (for heterodyne measurement), we can also obtain the details of the first comb line with central frequency Ω_m = 7.6 MHz. Signal to noise ratio of this line is also > 78 dB, verifying the stability. In **Fig. S12D**, we record the pulse train. Temporal period of this trace is 0.1316 μs, corresponding to the spectral interval 7.6 MHz. Pulse duration here is 20 ns, larger than the pulses shown in the maintext, because in this mode, *Q* factor is smaller.

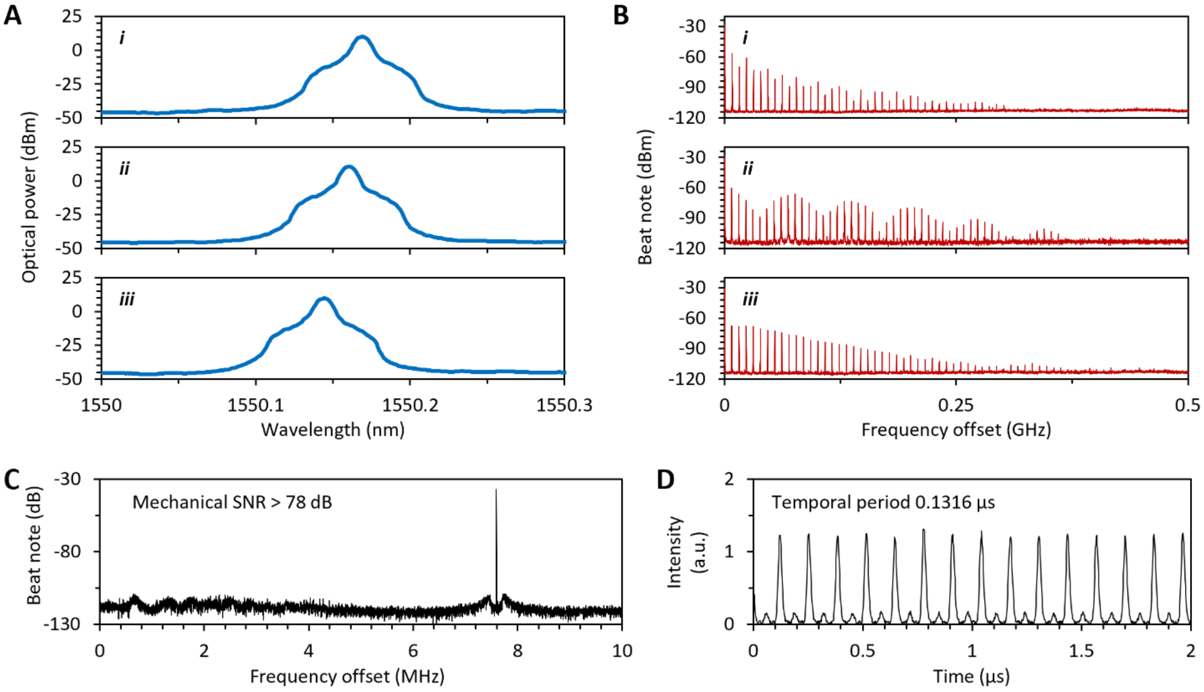

Fig. S12. Extended measurement of optomechanical oscillation states, without using an optical reference laser. (A) Optical spectra. **(B)** Radio frequency spectra. In **(A)** and **(B)**, we display three states (i-iii), they are transition state, two pulse locking state, and single pulse locking state. **(C)** Zoomed in spectrum of state (iii), here we see a high SNR line at 7.6 MHz. **(D)** Pulse train of the state (iii), demonstrates a period 0.1316 μs.

Figure S13 demonstrates extended measurements showing that we can deterministically generate mode-locked optomechanical combs across various optical resonances. In the main text, we verified that the formation of optomechanical mode locking in a graphene-based microresonator is universal; here, we provide additional examples. For instance, in **Fig. S13A**, we present results obtained by tuning the pump wavelength across four distinct, randomly selected modes, located at positions A-D. It is important to note that these four resonances are independent, without any mode crossing or splitting. The optical Q factors for these modes range from 4.9×10^7 to 1.02×10^8. Using a pump power of 400 mW, we achieve single soliton generation in all the modes. **Fig. S13B** plots the soliton spectra. Determined by the cavity geometry and material, the soliton repetition rate remains at 7.6 MHz. Moreover, we confirmed that the mode-locking state

can be achieved at resonances with various wavelengths. By utilizing a broadband tunable laser, we attempted exciting optomechanical oscillations at resonances of 1537.1 nm, 1543.8 nm, 1551.2 nm, 1562.3 nm, and 1573.4 nm (**Fig. S13C**). Due to graphene's gapless nature, saturable absorption effectively operates across a wide range, allowing us to achieve the optomechanical mode-locking state at diverse optical wavelengths, as shown in **Fig. S13D**. Here, Y means yes, N means no.

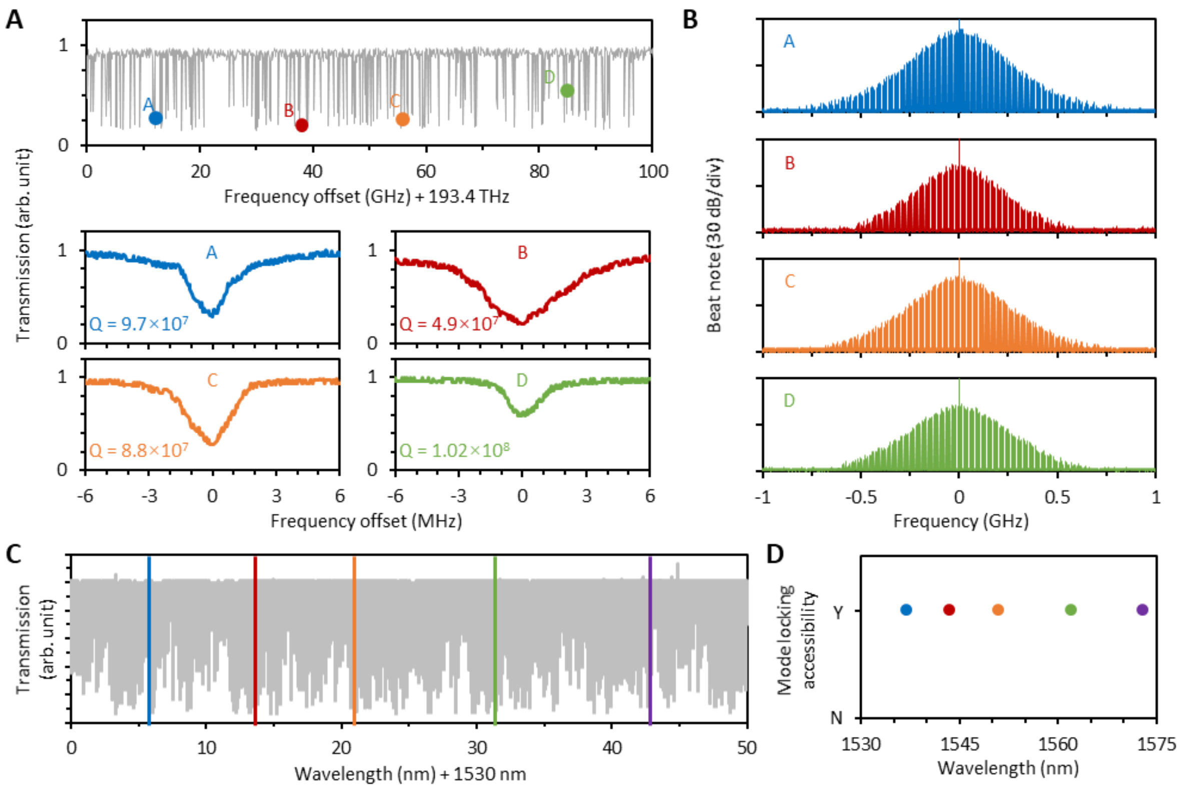

Fig. S13. Mode locking formation in varied optical mode families. (A) Transmission of our graphene based microcavity. In a complete FSR, there are 126 independent resonances. We can randomly select several resonances (A-D) for the generation of mode locked optomechanical oscillation. Here we also display the selected 4 resonances, with different optical *Q* factors. **(B)** Launching optical power into the selected 4 resonances, we successfully form mode locking states, here we demonstrate the spectra. (C) Exciting mode optomechanical oscillations in resonances with different wavelengths. (D) Mode locking state accessibility. For wavelength ranging from 1535 nm to 1575 nm, mode locking state could be achieved.

Figure S14 shows extended information that we use a radio frequency reference stabilizing the 62th comb line. **Fig. S14A** shows the spectrum of our radio frequency reference. The reference frequency is generated from a high precision signal generator (Model: R&S SMA100B, Option: SMAB-B711N). For beating with comb line, we set its frequency at 482.2 MHz. Linewidth of the electronic signal is < 1 Hz. **Fig. S14B** demonstrates the measured single sideband phase noise (SSB-PN) of this reference, suggesting that its SSB-PN approaches -93 dBc/Hz at 1 Hz offset, and -149.1 dBc/Hz at 1 MHz offset. When it stabilizes the 62th comb line, phase noise of the first comb line could be further suppressed to -110.9 dBc/Hz at 1 Hz offset, and -166.9 dBc/Hz at 1 MHz

offset in principles. In **Fig. S14C**, we illustrate its fast Allan deviation. For gating time from 1 μs to 1 s, frequency uncertainty of the reference signal decreases from 2.46×10^{-6} to 4.33×10^{-12}.

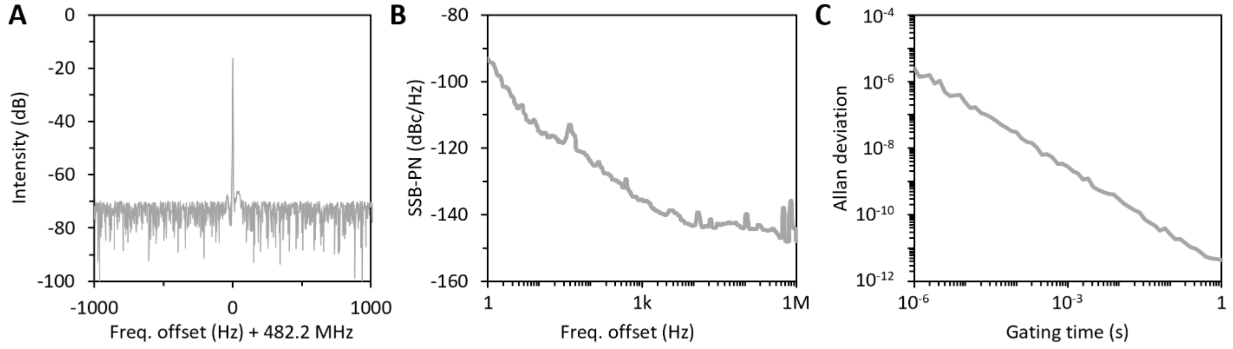

Fig. S14. Characterization of the radio frequency reference. (A) Spectrum. **(B)** Single sideband phase noise. **(C)** Allan deviation.

REFERENCES AND NOTES

1. T. J. Kippenberg, K. J. Vahala, Cavity optomechanics: Back-action at the mesoscale. *Science* **321**, 1172–1176 (2008).

2. M. Aspelmeyer, T. J. Kippenberg, F. Marquardt, Cavity optomechanics. *Rev. Mod. Phys.* **86**, 1391–1452 (2014).

3. S. Barzanjeh, A. Xuereb, S. Gröblacher, M. Paternostro, C. A. Regal, E. M. Weig, Optomechanics for quantum technologies. *Nat. Phys.* **18**, 15–24 (2022).

4. H.-K. Li, K. Y. Fong, H. Zhu, Q. Li, S. Wang, S. Yang, Y. Wang, X. Zhang, Valley optomechanics in a monolayer semiconductor. *Nat. Photonics* **13**, 397–401 (2019).

5. K. Stannigel, P. Rabl, A. S. Sørensen, P. Zoller, M. D. Lukin, Optomechanical transducers for long-distance quantum communication. *Phys. Rev. Lett.* **105**, 220501 (2010).

6. J. T. Hill, A. H. Safavi-Naeini, J. Chan, O. Painter, Coherent optical wavelength conversion via cavity optomechanics. *Nat. Commun.* **3**, 1196 (2012).

7. Y. Zhi, X.-C. Yu, Q. Gong, L. Yang, Y.-F. Xiao, Single nanoparticle detection using optical microcavities. *Adv. Mater.* **29**, 1604920 (2017).

8. D. Yu, M. Humar, K. Meserve, R. C. Bailey, S. N. Chormaic, F. Vollmer, Whispering-gallery-mode sensors for biological and physical sensing. *Nat. Rev. Methods Prim.* **1**, 83 (2021).

9. M. Wu, N. L.-Y. Wu, T. Firdous, F. Fani Sani, J. E. Losby, M. R. Freeman, P. E. Barclay, Nanocavity optomechanical torque magnetometry and radiofrequency susceptometry. *Nat. Nanotechnol.* **12**, 127–131 (2017).

10. Y. Arita, M. Mazilu, K. Dholakia, Laser-induced rotation and cooling of a trapped microgyroscope in vacuum. *Nat. Commun.* **4**, 2374 (2013).

11. G. A. Brawley, M. R. Vanner, P. E. Larsen, S. Schmid, A. Boisen, W. P. Bowen, Nonlinear optomechanical measurement of mechanical motion. *Nat. Commun.* **7**, 10988 (2016).

12. I. S. Grudinin, H. Lee, O. Painter, K. J. Vahala, Phonon laser action in a tunable two-level system. *Phys. Rev. Lett.* **104**, 083901 (2010).

13. J. Zhang, B. Peng, Ş. K. Özdemir, K. Pichler, D. O. Krimer, G. Zhao, F. Nori, Y. Liu, S. Rotter, L. Yang, A phonon laser operating at an exceptional point. *Nat. Photonics* **12**, 479–484 (2018).

14. T. Carmon, M. C. Cross, K. J. Vahala, Chaotic quivering of micron-scaled on-chip resonators excited by centrifugal optical pressure. *Phys. Rev. Lett.* **98**, 167203 (2007).

15. F. Monifi, J. Zhang, Ş. K. Özdemir, B. Peng, Y. Liu, F. Bo, F. Nori, L. Yang, Optomechanically induced stochastic resonance and chaos transfer between optical fields. *Nat. Photonics* **10**, 399–405 (2016).

16. J. Wu, S.-W. Huang, Y. Huang, H. Zhou, J. Yang, J.-M. Liu, M. Yu, G. Lo, D.-L. Kwong, S. Duan, C. Wei Wong, Mesoscopic chaos mediated by Drude electron-hole plasma in silicon optomechanical oscillators. *Nat. Commun.* **8**, 15570 (2017).

17. L. S. Cao, D. X. Qi, R. W. Peng, M. Wang, P. Schmelcher, Phononic frequency combs through nonlinear resonances. *Phys. Rev. Lett.* **112**, 075505 (2014).

18. A. Ganesan, C. Do, A. Seshia, Phononic frequency comb via intrinsic three-wave mixing. *Phys. Rev. Lett.* **118**, 033903 (2017).

19. G. Lin, Q. Song, Kerr frequency comb interaction with raman, brillouin, and second order nonlinear effects. *Laser Photon. Rev.* **16**, 2100184 (2022).

20. M.-A. Miri, G. D’Aguanno, A. Alù, Optomechanical frequency combs. *New J. Phys.* **20**, 043013 (2018).

21. J. Zhang, B. Peng, S. Kim, F. Monifi, X. Jiang, Y. Li, P. Yu, L. Liu, Y. Liu, A. Alù, L. Yang, Optomechanical dissipative solitons. *Nature* **600**, 75–80 (2021).

22. T. J. Kippenberg, A. L. Gaeta, M. Lipson, M. L. Gorodetsky, Dissipative Kerr solitons in optical microresonators. *Science* **361**, eaan8083 (2018).

23. A. Pasquazi, Optomechanics joins the soliton club. *Nat. Phys.* **17**, 1285–1286 (2021).

24. T. Herr, V. Brasch, J. D. Jost, C. Y. Wang, N. M. Kondratiev, M. L. Gorodetsky, T. J. Kippenberg, Temporal solitons in optical microresonators. *Nat. Photonics* **8**, 145–152 (2013).

25. H. Guo, M. Karpov, E. Lucas, A. Kordts, M. H. P. Pfeiffer, V. Brasch, G. Lihachev, V. E. Lobanov, M. L. Gorodetsky, T. J. Kippenberg, Universal dynamics and deterministic switching of dissipative Kerr solitons in optical microresonators. *Nat. Phys.* **13**, 94–102 (2017).

26. H. Zhou, Y. Geng, W. Cui, S.-W. Huang, Q. Zhou, K. Qiu, C. Wei Wong, Soliton bursts and deterministic dissipative Kerr soliton generation in auxiliary-assisted microcavities. *Light Sci Appl* **8**, 50 (2019).

27. L. Midolo, A. Schliesser, A. Fiore, Nano-opto-electro-mechanical systems. *Nat. Nanotechnol.* **13**, 11–18 (2018).

28. M. Romagnoli, V. Sorianello, M. Midrio, F. H. L. Koppens, C. Huyghebaert, D. Neumaier, P. Galli, W. Templ, A. D'Errico, A. C. Ferrari, Graphene-based integrated photonics for next-generation datacom and telecom. *Nat. Rev. Mater.* **3**, 392–414 (2018).

29. T. Tan, X. Jiang, C. Wang, B. Yao, H. Zhang, 2D material optoelectronics for information functional device applications: Status and challenges. *Adv. Sci.* **7**, 2000058 (2020).

30. A. Martinez, Z. Sun, Nanotube and graphene saturable absorbers for fibre lasers. *Nat. Photonics* **7**, 842–845 (2013).

31. C. Qin, K. Jia, Q. Li, T. Tan, X. Wang, Y. Guo, S.-W. Huang, Y. Liu, S. Zhu, Z. Xie, Y. Rao, B. Yao, Electrically controllable laser frequency combs in graphene-fibre microresonators. *Light Sci Appl* **9**, 185 (2020).

32. C. T. Phare, Y.-H. Daniel Lee, J. Cardenas, M. Lipson, Graphene electro-optic modulator with 30 GHz bandwidth. *Nat. Photonics* **9**, 511–514 (2015).

33. Z. Sun, A. Martinez, F. Wang, Optical modulators with 2D layered materials. *Nat. Photonics* **10**, 227–238 (2016).

34. B. Yao, Y. Liu, S.-W. Huang, C. Choi, Z. Xie, J. Flor Flores, Y. Wu, M. Yu, D.-L. Kwong, Y. Huang, Y. Rao, X. Duan, C. W. Wong, Broadband gate-tunable terahertz plasmons in graphene heterostructures. *Nat. Photonics* **12**, 22–28 (2018).

35. N. An, T. Tan, Z. Peng, C. Qin, Z. Yuan, L. Bi, C. Liao, Y. Wang, Y. Rao, G. Soavi, B. Yao, Electrically tunable four-wave-mixing in graphene heterogeneous fiber for individual gas molecule detection. *Nano Lett.* **20**, 6473–6480 (2020).

36. Y. Li, N. An, Z. Lu, Y. Wang, B. Chang, T. Tan, X. Guo, X. Xu, J. He, H. Xia, Z. Wu, Y. Su, Y. Liu, Y. Rao, G. Soavi, B. Yao, Nonlinear co-generation of graphene plasmons for optoelectronic logic operations. *Nat. Commun.* **13**, 3138 (2022).

37. O. Dogadov, C. Trovatello, B. Yao, G. Soavi, G. Cerullo, Parametric nonlinear optics with layered materials and related heterostructures. *Laser Photon. Rev.* **16**, 2100726 (2022).

38. V. Singh, S. J. Bosman, B. H. Schneider, Y. M. Blanter, A. Castellanos-Gomez, G. A. Steele, Optomechanical coupling between a multilayer graphene mechanical resonator and a superconducting microwave cavity. *Nat. Nanotechnol.* **9**, 820–824 (2014).

39. R. De Alba, F. Massel, I. R. Storch, T. S. Abhilash, A. Hui, P. L. McEuen, H. G. Craighead, J. M. Parpia, Tunable phonon-cavity coupling in graphene membranes. *Nat. Nanotechnol.* **11**, 741–746 (2016).

40. B. Yao, C. Yu, Y. Wu, S.-W. Huang, H. Wu, Y. Gong, Y. Chen, Y. Li, C. W. Wong, X. Fan, Y. Rao, Graphene-enhanced brillouin optomechanical microresonator for ultrasensitive gas detection. *Nano Lett.* **17**, 4996–5002 (2017).

41. J. Liu, F. Bo, L. Chang, C.-H. Dong, X. Ou, B. Regan, X. Shen, Q. Song, B. Yao, W. Zhang, C.-L. Zou, Y.-F. Xiao, Emerging material platforms for integrated microcavity photonics. *Sci. China Physics, Mech. Astron.* **65**, 104201 (2022).

42. B. Yao, S.-W. Huang, Y. Liu, A. K. Vinod, C. Choi, M. Hoff, Y. Li, M. Yu, Z. Feng, D.-L. Kwong, Y. Huang, Y. Rao, X. Duan, C. W. Wong, Gate-tunable frequency combs in graphene–nitride microresonators. *Nature* **558**, 410–414 (2018).

43. T. Tan, Z. Yuan, H. Zhang, G. Yan, S. Zhou, N. An, B. Peng, G. Soavi, Y. Rao, B. Yao, Multispecies and individual gas molecule detection using Stokes solitons in a graphene over-modal microresonator. *Nat Commun.* **12**, 6716 (2021).

44. F. Bonaccorso, Z. Sun, T. Hasan, A. C. Ferrari, Graphene photonics and optoelectronics. *Nat. Photonics* **4**, 611–622 (2010).

45. Y. Hu, S. Ding, Y. Qin, J. Gu, W. Wan, M. Xiao, X. Jiang, Generation of optical frequency comb via giant optomechanical oscillation. *Phys. Rev. Lett.* **127**, 134301 (2021).

46. H. Xiong, J. Gan, Y. Wu, Kuznetsov-Ma soliton dynamics based on the mechanical effect of light. *Phys. Rev. Lett.* **119**, 153901 (2017).

47. H. Xiong, Y. Wu, Optomechanical Akhmediev breathers. *Laser Photon. Rev.* **12**, 1700305 (2018).

48. Y. Guo, Z. Li, N. An, Y. Guo, Y. Wang, Y. Yuan, H. Zhang, T. Tan, C. Wu, B. Peng, G. Soavi, Y. Rao, B. Yao, A monolithic graphene functionalized microlaser for multispecies gas detection. *Adv. Mater.* **34**, 2207777 (2022).

49. C. Mo, J. Singh, J. R. Raney, P. K. Purohit, Cnoidal wave propagation in an elastic metamaterial. *Phys. Rev. E* **100**, 013001 (2019).

50. C. Wang, B. Chang, T. Tan, C. Qin, Z. Wu, G. Yan, B. Fu, Y. Wu, Y. Rao, H. Xia, B. Yao, High energy and low noise soliton fiber laser comb based on nonlinear merging of Kelly sidebands. *Opt. Express* **30**, 23556–23567 (2022).

51. S. Sun, B. Wang, K. Liu, M. W. Harrington, F. Tabatabaei, R. Liu, J. Wang, S. Hanifi, J. S. Morgan, M. Jahanbozorgi, Z. Yang, S. M. Bowers, P. A. Morton, K. D. Nelson, A. Beling, D. J. Blumenthal, X. Yi, Integrated optical frequency division for microwave and mmWave generation. *Nature* **627**, 540–545 (2024).

52. Y. Zhao, J. K. Jang, G. J. Beals, K. J. McNulty, X. Ji, Y. Okawachi, M. Lipson, A. L. Gaeta, All-optical frequency division on-chip using a single laser. *Nature* **627**, 546–552 (2024).

53. B. C. Yao, W. T. Wang, Z. Da Xie, Q. Zhou, T. Tan, H. Zhou, G. C. Guo, S. N. Zhu, N. H. Zhu, C. W. Wong, Interdisciplinary advances in microcombs: Bridging physics and information technology. *eLight* **4**, 19 (2024).

54. R. Wu, B. Chen, D. Liu, G. Qiu, Z. Liu, D. Wei, J. Liu, Revealing resonant mode properties in asymmetric photonic crystal microrings through diverse excitation methods. *Nano Lett.* **25**, 9501–9507 (2025).

55. Y. Wang, M. Zhang, Z. Shen, G.-T. Xu, R. Niu, F.-W. Sun, G.-C. Guo, C.-H. Dong, Optomechanical frequency comb based on multiple nonlinear dynamics. *Phys. Rev. Lett.* **132**, 163603 (2024).

56. A. G. Krause, J. T. Hill, M. Ludwig, A. H. Safavi-Naeini, J. Chan, F. Marquardt, O. Painter, Nonlinear radiation pressure dynamics in an optomechanical crystal. *Phys. Rev. Lett.* **115**, 233601 (2015).

57. Q. Bao, H. Zhang, Y. Wang, Z. Ni, Y. Yan, Z. X. Shen, K. P. Loh, D. Y. Tang, Atomic-layer graphene as a saturable absorber for ultrafast pulsed lasers. *Adv. Funct. Mater.* **19**, 3077–3083 (2009).

58. X. Yi, Q.-F. Yang, K. Y. Yang, M.-G. Suh, K. Vahala, Soliton frequency comb at microwave rates in a high-Q silica microresonator. *Optica* **2**, 1078 (2015).

59. X. Yi, Q.-F. Yang, K. Y. Yang, K. Vahala, Theory and measurement of the soliton self-frequency shift and efficiency in optical microcavities: Publisher's note. *Opt. Lett.* **41**, 3722 (2016).

60. K. J. A. Ooi, D. T. H. Tan, Nonlinear graphene plasmonics. *Proc. R. Soc. A Math. Phys. Eng. Sci.* **473**, 20170433 (2017).

61. A. Marini, J. D. Cox, F. J. García De Abajo, Theory of graphene saturable absorption. *Phys. Rev. B* **95**, 1–11 (2017).

62. Q. Bao, H. Zhang, Z. Ni, Y. Wang, L. Polavarapu, Z. Shen, Q.-H. Xu, D. Tang, K. P. Loh, Monolayer graphene as a saturable absorber in a mode-locked laser. *Nano Res* **4**, 297–307 (2011).

63. R. R. Nair, P. Blake, A. N. Grigorenko, K. S. Novoselov, T. J. Booth, T. Stauber, N. M. R. Peres, A. K. Geim, Fine structure constant defines visual transparency of graphene. *Science* **320**, 1308–1308 (2008).
